# A systems biology approach reveals neuronal and muscle developmental defects after chronic exposure to ionising radiation in zebrafish

**DOI:** 10.1038/s41598-019-56590-w

**Published:** 2019-12-27

**Authors:** Sophia Murat El Houdigui, Christelle Adam-Guillermin, Giovanna Loro, Caroline Arcanjo, Sandrine Frelon, Magali Floriani, Nicolas Dubourg, Emilie Baudelet, Stéphane Audebert, Luc Camoin, Olivier Armant

**Affiliations:** 1 Institut de Radioprotection et de Sûreté Nucléaire (IRSN), PSE-ENV/SRTE/LECO, Cadarache, Saint-Paul-lez-Durance, 13115 France; 2 Aix-Marseille University, Inserm, CNRS, Institut Paoli-Calmettes, CRCM, Marseille Proteomics, Marseille, France; 3 Institut de Radioprotection et de Sûreté Nucléaire (IRSN), PSE-SANTE/SDOS/LMDN, Cadarache, Saint-Paul-lez-Durance, 13115 France

**Keywords:** Embryogenesis, Transcriptomics, Systems analysis

## Abstract

Contamination of the environment after the Chernobyl and Fukushima Daiichi nuclear power plant (NPP) disasters led to the exposure of a large number of humans and wild animals to radioactive substances. However, the sub-lethal consequences induced by these absorbed radiological doses remain understudied and the long-term biological impacts largely unknown. We assessed the biological effects of chronic exposure to ionizing radiation (IR) on embryonic development by exposing zebrafish embryo from fertilization and up to 120 hours post-fertilization (hpf) at dose rates of 0.5 mGy/h, 5 mGy/h and 50 mGy/h, thereby encompassing the field of low dose rates defined at 6 mGy/h. Chronic exposure to IR altered larval behaviour in a light-dark locomotor test and affected cardiac activity at a dose rate as low as 0.5 mGy/h. The multi-omics analysis of transcriptome, proteome and transcription factor binding sites in the promoters of the deregulated genes, collectively points towards perturbations of neurogenesis, muscle development, and retinoic acid (RA) signaling after chronic exposure to IR. Whole-mount RNA *in situ* hybridization confirmed the impaired expression of the transcription factors *her4.4* in the central nervous system and *myogenin* in the developing muscles of exposed embryos. At the organ level, the assessment of muscle histology by transmission electron microscopy (TEM) demonstrated myofibers disruption and altered neuromuscular junctions in exposed larvae at 5 mGy/h and 50 mGy/h. The integration of these multi-level data demonstrates that chronic exposure to low dose rates of IR has an impact on neuronal and muscle progenitor cells, that could lead to motility defects in free swimming larvae at 120 hpf. The mechanistic understanding of these effects allows us to propose a model where deregulation of RA signaling by chronic exposure to IR has pleiotropic effects on neurogenesis and muscle development.

## Introduction

Living organisms have evolved robust genetic networks to detect DNA damage induced by genotoxic stress and to arrest the cell cycle, allowing time for repairing the damages. The induction of DNA damage either generated from reaction with byproducts of the metabolism or induced by external genotoxic stressors such as ionizing radiations (IR) must be tightly controlled to avoid profound impacts on cell functions. Large scale contaminations of the environment after the Chernobyl and Fukushima Daiichi nuclear power plant (NPP) disasters exposed a large number of humans and wild animals to radioactive substances^1,2^. If the acute exposure phase following these releases led to rather well characterized effects, the sub-lethal effects due to chronic exposures to IR remain understudied and hence, the long-term biological impacts largely unknown. Studies on wild animals reported that brains of birds and monkey fetuses from Chernobyl^3^ and Fukushima Daiichi NPP^4^ areas respectively, have a smaller size. In addition, epidemiological studies on children born from Hiroshima and Nagasaki survivors demonstrated brain development defects^5^ and reduced cognitive capacities^6^, for doses of IR as low as 0.31 Gy^7^.

IR induces DNA double stranded breaks (DSBs) that are detrimental for cell survival if not repaired. DSBs lead to the phosphorylation of the histone variant H2AX on serine 139 by the phospho-inositol-3 kinase ATM and the formation of γ-H2AX nuclear foci at the sites of DSBs that are detectable by specific antibodies, thereby providing a simple assay to assess genotoxicity. Studies on the effects of low dose rates of IR (< 6 mGy/h)^8^ on rodents are contradictory as genotoxic effects could be observed in some studies but not in others^9–12^. In addition, these studies were performed on adult organisms which are more resilient to genotoxic stressors than developing embryos. A study on zebrafish embryos exposed at 0.03 mGy/h and 24 mGy/h showed an acceleration of hatching rate, induction of oxidative stress and DNA damage^13^. In another study zebrafish eggs were exposed at 0.54 to 10.9 mGy/h and analysed by high-throughput sequencing at early gastrulation stage, near 5.5 hours post fertilization (hpf)^14^. Changes in important developmental pathways such as lateral inhibition (Notch signaling) and retinoic acid (RA) pathway (involved in anteroposterior patterning) were observed in early gastrula embryos, but a direct assessment of the consequences of these early changes at later developmental stages was not assessed.

The complex gene regulatory networks active in all multicellular organisms are the product of transcriptional regulators interacting with cis-regulatory modules (CRM) like promoters, enhancers, silencers and locus control regions that, together with epigenetics processes, regulate the expression of genes. Molecular changes obtained from transcriptomics and proteomics data are widely used in system biology approaches to generate hypothesis on the physiological effects observed at higher organisational levels, as well as for the identification of biomarkers. However, as cell-signaling target ultimately transcription factors (TF) to modify gene expression, identifying TF that drives changes in gene expression may reveal the key signaling pathways during disease progression. To regulate transcription, TF bind specific DNA sequences in the promoter of the target genes, referred as transcription factor binding sites (TFBS). The enrichment of TFBS in the promoters of the co-expressed genes can be used to predict the TF that drive changes in gene expression and ultimately reveal the underlying regulatory mechanisms of disease progression^15–17^. In this context, establishing links between molecular and physiological alterations leading to adverse effects such as lethality, carcinogenesis or behavioural alterations, fits very well with the concept of adverse outcome pathway (AOP) which establishes causal relationships between an initiating molecular event and the adverse effects at a biological level relevant for risk assessment^18,19^.

In the present study we focused on the *in vivo* effects of low to moderate dose rates of IR on embryonic development using zebrafish embryos, a model commonly used in toxicogenomics, disease modelling and developmental biology^20–22^. We assessed the biological effects of chronic exposure of low to moderate dose rates of IR at different levels of organisation from molecular alterations to higher-order alterations such as behaviour. Morphological and functional measurements were performed at various developmental stages, from 24 hpf to 120 hpf larvae and after exposure at three different dose rates of gamma radiation ranging from 0.5 mGy/h to 50 mGy/h. We assessed the potential effects on behaviour by measuring embryonic activity at 24 hpf and performed a visual motor response test on 120 hpf larvae. At the molecular levels we performed a multi-OMICS analysis by studying the effects of IR on both the transcriptome and the proteome. Analysis of the TFBS in the promoters of the deregulated genes pin-points perturbations of key TF in embryonic development. Changes in gene expression were further confirmed by whole mount RNA *in situ* hybridization, and histology assessed by transmission electron microscopy (TEM).

## Results

### Chronic exposure to low dose rates of IR increases DNA damages and alters behaviour

**Figure 1:**
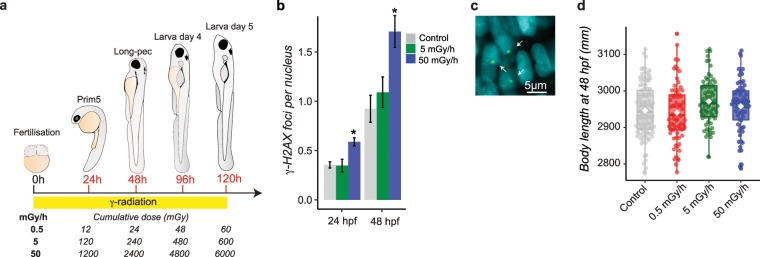
(**a**) Experimental design and dosimetry. The cumulative dose in Gy are indicated for each developmental stages and sampling time (in red). (**b**) Detection of γ-H2AX foci in 24 hpf and 48 hpf embryos by whole-mount immunocytochemistry. Standard-error to the mean is indicated for each exposure (Kruskall-wallis test followed by a pairwise Wilcoxon rank sum post-hoc test and adjusted by the Holm method, adjusted p-value: * < 0.05). (**c**) Examples of positive γ-H2AX foci (green, white arrows) detected in DAPI stained nucleus (blue) by confocal microscopy. (**d**) Means of body length measured at 48 hpf (in mm) are indicated as white lozenge. Dots represent individual data.

We challenged zebrafish embryos to chronic exposures of IR from early developmental stage (1 hpf) up to late larval stage (120 hpf) (Fig. 1a). Acute exposures to high doses of gamma rays increase the occurrence of developmental defects^23^ and induce DSBs in zebrafish cells^24^. DSBs are difficult to repair and may lead to irreversible effects such as developmental impairments and cancer. To assess these adverse effects, we analysed γ-H2AX foci by whole-mount immunostaining in 24 hpf embryos and 48 hpf larvae and studied embryonic development over a 96 h period. We detected a significant increase of γ-H2AX foci in 24 hpf embryos and 48 hpf larvae for the highest dose (adjusted p-value < 10^−3^ and < 0.01 respectively), but no significant change was observed at lower dose rates (Fig. 1b,c). Other macroscopic endpoints such as embryonic lethality, hatching success and body length were largely unaffected (Table 1, Fig. 1d), which suggests that, in the conditions tested here, chronic exposure to 50 mGy/h of IR induces DNA damages without affecting embryonic survival.

**Table 1:** Percentage (%) of Embryonic mortality at 24 hpf and hatching rate at 72 hpf with the corresponding 95% confidence intervals (CI) indicated in brackets.

Dose rate (mGy/h)	% Survival rate at 24 hpf (95% CI)	% Hatching rate at 72 hpf (95% CI)
Control	84.9 (80.6–88.4)	100 (98.4–100)
0.5	91.3 (85.5–95.0)	100 (96.8–100)
5	88.1 (81.8–92.5)	100 (96.7–100)
50	82.1 (75.6–87.2)	99.3 (95.8–100)

**Figure 2:**
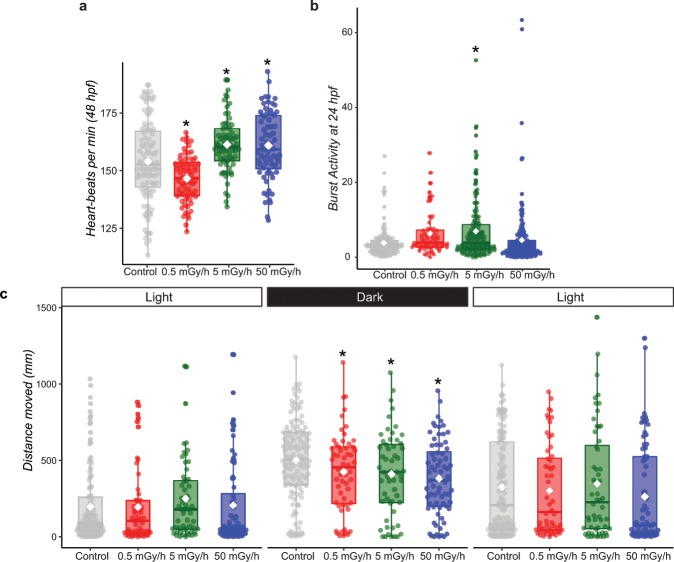
(**a**) Box-plot of cardiac activity shown as heart beats per minute in 48 hpf larvae. Dots represent individual data. Means are indicated as white lozenge (Kruskall-wallis test followed by a pairwise Wilcoxon rank sum post-hoc test and adjusted by the Holm method, adjusted p-value: * < 0.05). (**b**) Box-plot of embryonic burst activity at 24 hpf. Means are indicated as white lozenge (significance of permutation test p-value: * < 0.05). (**c**) Box-plot of larval motility with the visual motor response test 120 hpf followed over three consecutive light cycles of 5 min (indicated at the top). The mean distance travelled by larvae is indicated as white lozenge. Results of a permutation test are indicated (* p-value < 0.05).

We next assessed if chronic exposure to IR could affect cardiac activity and behaviour, two physiologically integrated parameters proposed to be more sensitive than macroscopic endpoints^25^. Cardiac activity measured in 48 hpf larvae showed a complex non-linear response to the dose rate, as we observed a significant decrease of cardiac beats per minute at the lowest dose rate (0.5 mGy/h, adjusted p-value < 0.05, n = 69), and an increase at the two higher dose rates (5 mGy/h adjusted p-value < 0.05, n = 69; 50 mGy/h: adjusted p-value < 0.05, n = 68) (Fig. 2a). Zebrafish embryonic activity is heavily used to assess chemical toxicity, as it is easily done^26,27^. Such assays detect embryonic burst activity, which correspond to the number of spontaneous tail-coiling of 24 hpf embryos. Twenty-four hour-long chronic exposure to low dose rate of IR induced a significant increase (p-value < 0.05, n = 156) of embryonic activity at 5 mGy/h, but not at 0.5 mGy/h nor at the highest dose rate of 50 mGy/h (Fig. 2b). This effect was retrieved consistently in three independent experiments which indicates that this non-linear dose-response is robust. We next measured larval motility at 120 hpf under stress conditions^28^. In this visual motor response test, larvae are exposed to three sharp changes of light intensity to elicit a stress response. A significant decrease of larval motility was observed in the dark phase at the three dose rates (permutation test, p-value < 0.05, Fig. 2c), which suggests that exposures to IR can alter zebrafish escape behaviour at a dose rate as low as 0.5 mGy/h.

### Transcriptomics analysis reveals impairment of major developmental pathway after chronic exposure to IR

**Figure 3:**
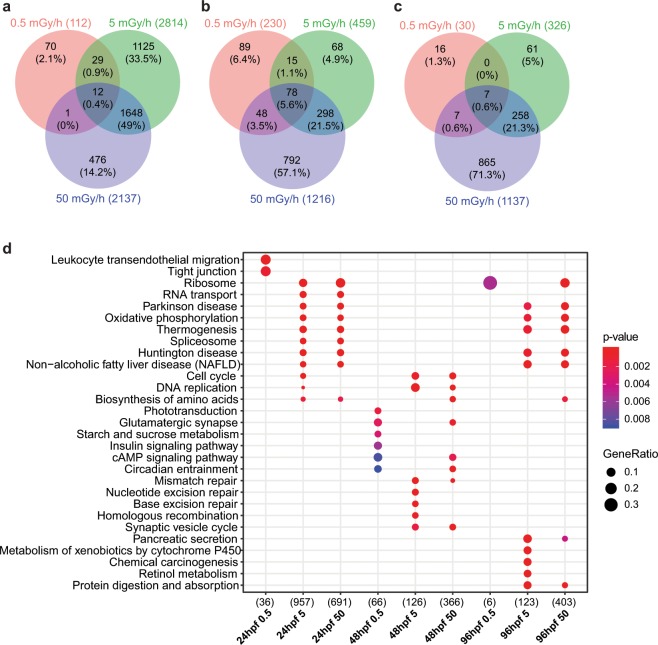
Venn diagram of differentially expressed genes at the three dose rates (|fold change| ≥ 1.5 and adjusted p-value < 0.01), at (**a**) 24 hpf, (**b**) 48 hpf and (**c**) 96 hpf. The number of up or down-regulated genes are indicated in brackets. (**d**) Dot plot of KEGG enrichment with human orthology showing the top-enriched pathways. The total numbers of deregulated genes within the KEGG pathways selected on the dot plot are indicated in brackets. Colours indicate the enrichment p-values from exact Fisher’s test, and dots size is proportional to the number of genes constituting the given pathway.

Transcriptomics analysis was performed in order to identify the genetic pathways altered upon chronic exposure to low doses of IR. Global analysis of the transcriptome was performed at 24 hpf, 48 hpf and 96 hpf to follow the dynamics of gene expression during zebrafish development. A total of 5.7 billion of good quality reads (Q > 30) was produced and differential gene expression assessed in pairwise comparison between the embryos or larvae exposed at 0.5 mGy/h, 5 mGy/h or 50 mGy/h and the stage specific controls. The total number of differentially expressed genes (DEG) within our significance threshold (|fold change| ≥ 1.5 and adjusted p-value < 0.01) decreased with the developmental stage (the complete list of significantly deregulated genes is provided in Supplementary Table T4). In addition, fewer genes were differentially expressed for the lower dose rate (0.5 mGy/h) compared to the two highest dose rates (5 mGy/h and 50 mGy/h) (Fig. 3a–c). The quality of mRNAseq data was checked by TaqMan quantitative RT-PCR; the Pearson’s correlation coefficients between the two methods (ρ > 0.6) were consistent with previous studies (Supplementary Fig. S3)^29,30^. Very few genes were found to be mis-regulated in common between the three dose rates at any of the three stages studied: 12 genes in 24 hpf embryos, 78 genes in 48 hpf larvae and 7 genes in 96 hpf larvae (Fig. 3a–c). The highest overlaps were observed consistently for the two highest dose rates (21% to 49%), indicating that the transcriptional responses under these two conditions were more similar compared to the lower dose-rate.

Searching shared functions among different conditions is a common way to incorporate the biological knowledge provided by biological ontologies. We performed pathways enrichment analysis using the KEGG (with human orthologous genes) and GO repositories (with zebrafish and human orthologues) and found that the most deregulated pathways at the two higher dose rates were related to neurological disorders, cell cycle, neurogenesis and synaptic transmission (Fig. 3d, Supplementary Fig. S4 and S5). A complete list of zebrafish GO terms enriched in each condition is provided in Supplementary Table T5. Key developmental processes such as neurogenesis (GO:0050767, Fisher’s exact test p-value < 1.10^−4^), somite development (GO:0061053, p-value < 1.10^−5^), retinol metabolism (GO:0042572, p-value < 0.01) and hematopoiesis (GO:0048534, p-value < 1.10^−4^) were impacted at the two highest dose rates (50 mGy/h and 5 mGy/h). Very few biological pathways were generally enriched at 0.5 mGy/h in 48 hpf and 96 hpf larvae due to the small number of DEG detected in these conditions, but we detected a significant enrichment of cell cycle (GO:0045786, p-value < 0.01) and blood vessels development (GO:0097496, p-value < 0.01) in 24 hpf embryos. The enriched GO terms for the 78 DEG mis-regulated in common for the 3 dose rates at 48 hpf (which corresponds to the highest overlap, Fig. 3b), were composed of genes involved in neuron development (GO:0048666, p-value < 0.01), steroid hormone mediated signalling pathway (GO:0043401, p-value < 0.01) and programmed cell death (GO:0010623, p-value < 0.01) (Supplementary Table T6). The protein chaperons *hsp70l* and *hspb1* involved in stress response, were upregulated at 24 hpf at 5 and 50 mGy/h. The detailed analysis of gene expression in the different conditions showed a deregulation of developmental processes at different phases of cell specification and differentiation. For instance, *myog*, *lef1*, *tbx16*, *gata6* and *rdh10b*, expressed during the early phase of embryonic development but not at later stage, were mis-regulated at 24 hpf for the two highest dose rates, while *elavl4* and *cux1*, involved later in the neuronal differentiation process, were altered at 48 hpf and 96 hpf (Supplementary Fig. S6).

### Deregulation of transcription factors expression after exposure to chronic irradiation

**Figure 4:**
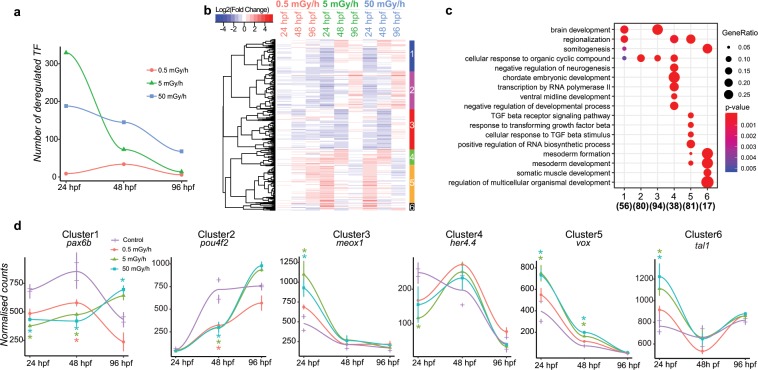
(**a**) Number of transcription factors (TF) with significant deregulation (|fold change| ≥ 1.5 and adjusted p-value < 0.01) over developmental time at three different dose rates. (**b**) Hierarchical clustering of 462 TF misregulated in at least one comparative analysis. Blue indicate downregulation, white no change and red upregulation, as compared to control conditions. Clusters of co-regulated genes are indicated at the right from 1 to 6. (**c**) GO enri**c**hment of TF classified in 6 different clusters. Cluster number are indicated below, the total number of deregulated TF in each GO term in brackets. (**d**) Expression patterns (as normalised count) of key master regulators during development using loess smoothed conditional means. Cluster numbers are indicated at the top. *|fold change| ≥ 1.5 and adjusted p-value < 0.01.

TF are key regulators that orchestrate the developmental program in a time and tissue specific manner. TF were categorized into families based on their DNA-binding domains (see Material and Methods section and Supplementary Table T3) and their differential expression assessed over developmental time at the three dose rates of IR (Fig. 4a). The number of TF significantly deregulated after exposure to IR was higher in 24 hpf embryos and decreased in the subsequent developmental stages at 5 mGy/h and 50 mGy/h. At the lower dose rate of 0.5 mGy/h, the number of mis-regulated TF peaked at 48 hpf and remained always lower compared to the two other conditions. We then assessed the expression pattern of the 462 TF displaying a significant change in at least one condition by hierarchical clustering. The expression profiles of TF were very similar over developmental time for the exposures at 5 and 50 mGy/h (Fig. 4b). Fewer TF displayed an altered expression profile at 0.5 mGy/h compared to the higher dose rates, as observed before in the global gene expression analysis. Six different clusters were identified based on gene expression pattern. Cluster 1, 2 and 3 were composed of TF downregulated at 24 hpf or 48 hpf, and upregulated or unchanged at 96 hpf. TF belonging to clusters 4, 5 and 6 were in contrast strongly upregulated at 24 hpf and then mostly not changed or upregulated at 48 hpf and 96 hpf (Fig. 4b). Gene Ontology enrichments demonstrated a significant enrichment of TF involved in brain development, somitogenesis and response to cyclic organic compound in all 6 clusters (Fig. 4c). Expression patterns of key master regulators of neurogenesis such as *pax6b*, *her4.4* and *pou4f2*, and mesoderm development, *meox1*, *tal1* and *vox* were impaired (Fig. 4d). Many of these TF were highly expressed at 24 hpf or 48 hpf, but had little or no expression at 96 hpf (*pax6b*, *meox1*, *her4.4*, *vox* on Fig. 4d), consistent with their role during early cell specification processes. An analysis of the 1084 genes involved in the neurogenesis process (GO:0022008) confirmed that many neuronal genes were mis-regulated after chronic exposure, especially at 5 mGy/h and 50 mGy/h (Supplementary Fig. S7). Together, these data suggest that the developmental programs regulating muscle and central nervous system development are impaired in embryos and larvae exposed to 5 and 50 mGy/h, and to a lesser extent at 0.5 mGy/h.

### TF binding sites analysis and proteomics coherently highlight impacts on neurogenesis and muscle development

**Figure 5:**
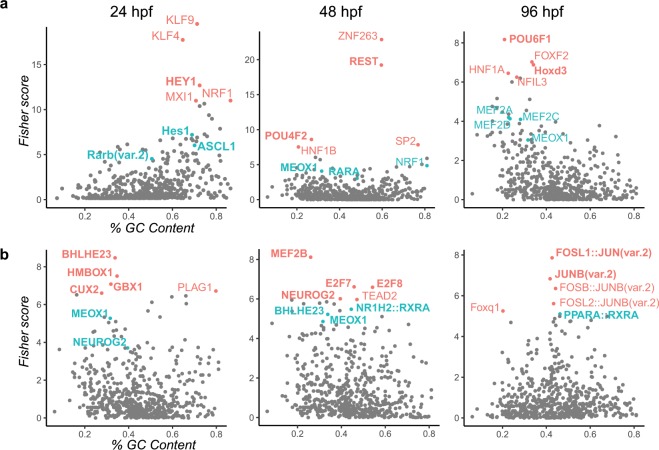
(**a**) Transcription factor binding sites enrichment in the promoters of upregulated genes, and (**b**) in downregulated genes at 24 hpf, 48 hpf and 96 hpf, after exposure to 50 mGy/h. Top 5 enriched transcription factor binding sites are indicated in red, and other binding sites of interest, near the limit of significance, in blue. Master regulators involved in neurogenesis, myogenesis, synaptogenesis and RA pathways are highlighted in bold.

The global analysis of expression data suggests that the development of the central nervous system and the muscles of embryos exposed to chronic irradiation are impaired. During embryogenesis, master transcriptional regulators orchestrate the expression dynamic of many genes and are central in the regulation of gene regulation networks. Genes with altered expression can be used to predict the master transcriptional regulators that drive changes in gene expression^17^, thereby pin-pointing the key transcriptional processes affected by chronic exposure to IR. To do so, we analysed the promoter of all deregulated genes at 24 hpf, 48 hpf and 96 hpf, focusing on the highest dose rate of 50 mGy/h as this exposure leads to many DEG (required for such promoter analysis) and displays very similar transcriptomics responses compared to 5 mGy/h. We found that DNA binding sites of key TF involved in neurogenesis (HEY1, HES1, REST, ASCL1, NEUROG2, POU4F2), synaptogenesis (MEF2 family) and myogenesis (MEOX1) were highly enriched in the promoters of DEG at all stages (Fig. 5). We also noted that retinoic acid responsive elements (RARE), specific to the RA pathway, were also enriched in the promoters of DEG (Fig. 5). To confirm potential binding of TF on the promoter of DEG, we compared the list of potential transcriptional targets identified for HES1 and MEF2 in our study, to the ChIP-Seq data availed in ENCODE project^31^. From the initial 865 DNA-binding sites identified *in silico* for HES1 in our promoter analysis, 166 (20%) were *bona fide* transcriptional targets detected by ChIP-Seq experiments. Similarly, 79 out of 311 binding sites identified *in silico* (25%) were also detected in MEF2B ChIP-Seq data.

**Figure 6:**
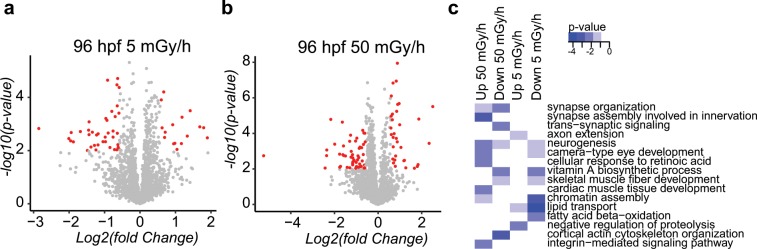
Volcano plots of deregulated proteins in 96 hpf larvae exposed (**a**) at 5 mGy/h and (**b**) at 50 mGy/h. Proteins with significant expression change (|fold change| ≥ 1.5 and p-value < 0.01) after IR exposure are highlighted in red. (**c**) Heatmap of GO terms enriched in the proteomics data. Significant p-values (Fisher’s exact test) are in blue (< 0.01) and non-significant p-value in white.

To further characterise the impacts of chronic irradiation stress observed at the transcriptomics scale, we performed a global analysis at the protein level by a proteomics analysis. This analysis was focused on the 96 hpf larvae exposed at the two highest dose rates, as the required protein quantities were limiting for the earlier developmental stages and the 0.5 mGy/h conditions had only mild effects (based on the transcriptomics data). We found respectively 56 (Supplementary Table T7) and 88 (Supplementary Table T8) differentially expressed proteins in 96 hpf larvae exposed at 50 mGy/h and 5 mGy/h (Fig. 6a,b). The molecular pathways significantly enriched in the proteomics data (p-value from Fisher’s exact test < 0.01) were all related to neurogenesis, somitogenesis, synapse organisation and vitamin A metabolic processes (Fig. 6c). We could not find any direct significant correlations between proteins and mRNA expression (data not shown), as already described in previous multi-omics data^32^.

### Alteration of gene expression patterns and disruption of muscle ultrastructure

**Figure 7:**
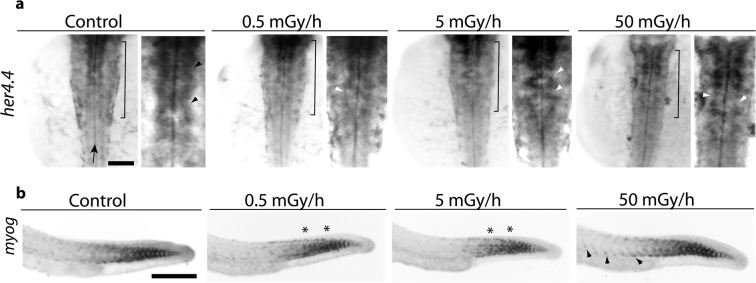
Whole mount RNA *in situ* hybridization on 24 hpf embryos. (**a**) Dorsal view of embryos oriented with anterior (head) towards to the top. Right pictures are higher magnifications of area indicated in the rhombencephalon by the black brackets. Black arrow indicates the position of the central canal. Black arrow heads indicate the position of *her4.4* positive neuronal progenitors flanking the central in control embryos and white arrow heads missing progenitors in exposed embryos. (**b**) Lateral view of 24 hpf embryos labelled with *myog*, anterior to the left. The black arrow heads indicate ectopic expression of *myog* in somites located in the anterior part of the trunk at 50 mGy/h. * Indicates the position of mis-shaped somites in the trunk. Scale bares: 200 µm.

As the molecular data points coherently towards impairments of neurogenesis and muscle development after exposure to low dose rates of IR, we investigated if progenitor cell populations could be altered in the developing central nervous system (neural tube) and muscles (called somites in embryos) of zebrafish embryos. Whole mount RNA *in situ* hybridization analysis was produced on 24 hpf embryos (n = 10), a stage at which both neuronal and muscle progenitors are present. At 24 hpf, *her4.4* labels two strips of neuronal progenitors flanking the central canal in the hindbrain (the most posterior part of the anterior forebrain). We observed gaps and irregular expression of *her4.4* along the central canal in the hindbrain of exposed embryos at the three dose rates (Fig. 7a), which is consistent with the downregulation of *her4.4* observed by mRNAseq at 24 hpf (Fig. 4d). Differentiating muscle progenitors are labelled in control embryos by *myog* in a well-defined chevron-shape pattern characteristic of developing somites. After exposure to 50 mGy/h, we detected ectopic *myog* positive cells located more anteriorly in the trunk compared to control (Fig. 7b). We could not detect such ectopic expression at 5 mGy/h and 0.5 mGy/h, but observed irregular expression of *myog* (asterisks in Fig. 7b) which suggests that somites have lost their classical chevron-shape.

**Figure 8:**
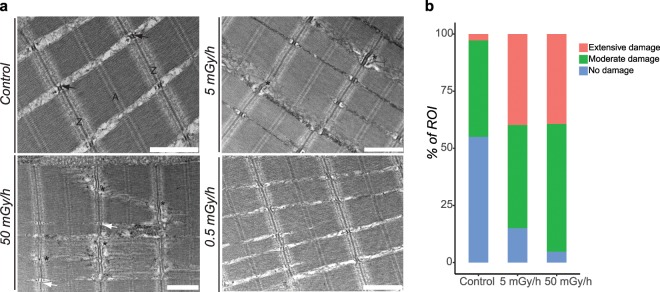
(**a**) Transmission electron microscopy of muscles in 96 hpf larvae. A and Z bands of sarcomeres are indicated. * Indicates altered myofibers. Arrows indicate the triads constituted of t-tubes and the two flanking cisterna of the sarcoplasmic reticulum (black: normal, white: deformed). Scale bares: 1 µm. (**b**) Categorization and quantification of myofiber damage. The percentage of TEM images defined as ROI (region of interest) displaying normal or damaged (moderate or extensive) myofibers was computed from at least 21 independent ROI in triplicate (n > 71 for each group).

We then performed an analysis of the muscle fibres and neuromuscular junctions at later developmental stage by transmission electron microscopy (TEM), in 96 hpf larvae. Sarcomeres, the structural unit of muscles, are organized in a regular succession of A and Z-bands in control larvae. In addition, we could detect well-defined t-tubules and the two flanking cisterna of the sarcoplasmic reticulum (Fig. 8a). In contrast, a frequent swelling of the Z-bands and detachments of the myofibers were detected in larvae exposed at 50 mGy/h and 5 mGy/h (Fig. 8a). In addition the cisterna of the sarcoplasmic reticulum were not well shaped at 50 mGy/h but looked normal at 5 mGy/h (Fig. 8a). No effects were detected in the striated muscles at 0.5 mGy/h. Myofiber damages observed by TEM were classified into three categories: no damage, moderate or extensive damages. A significantly higher proportion of damaged myofibers (Pearson’s Chi-squared test adjusted by the Holm method, adjusted p-value < 10^−10^) was detected by TEM in larvae exposed to 5 mGy/h and 50 mGy/h compared to control larvae (Fig. 8b).

## Discussion

We analysed the biological effects of chronic exposure to IR at 0.5 mGy/h, 5 mGy/h and 50 mGy/h on zebrafish development at multiple levels of biological organisation. At the individual level, we observed a decrease of larval motility at 120 hpf at all dose rates during the dark phase of the larval motility test. Previous studies suggested that locomotion is strongly increased during this phase, allowing the detection of fine effects on photokinesis driven by deep-brain photoreceptors^33,34^. In addition to this effect on larval locomotion, we found subtle but significant changes in embryonic and cardiac activities at 24 hpf and 48 hpf respectively. By using TEM analysis on the striated muscles of 96 hpf larvae, we could observe myofiber disruptions and malformed sarcoplasmic reticulum at 5 mGy/h and 50 mGy/h. Muscle contraction being dependent on functional sarcoplasmic reticulum, these results suggest that neuromuscular functions could be impaired after exposure to IR at 5 mGy/h and 50 mGy/h. In favour of such hypothesis, the transcriptomics data showed that zebrafish genes mediating Ca^2+^ release in the sarcoplasmic reticulum, such as *ryr3* and *tnnt2d* were significantly downregulated (fold change < 2 and adjusted p-value < 0.01) in 24 hpf embryos at 5 mGy/h and 50 mGy/h. An alteration of myosin filaments at 0.03 mGy/h and 23.75 mGy/h has already been observed after exposure of zebrafish embryos^13^, which is in accordance with our results that show disruption of myofibers by TEM. However, the direct links between altered neuromuscular junctions, somitogenesis defects and decreased larval locomotion remain to be clearly established.

At the molecular and cellular levels, we found that the biological processes of neurogenesis and muscle development were affected at dose rates as low as 0.5 mGy/h. In a precedent transcriptomics analysis of acute exposure of 26 hpf zebrafish embryos to high dose of IR (> 1 Gy), Freeman *et al*. showed misregulation of few genes involved in neurogenesis and cardiovascular development^23^. In the field of low dose rates, a recent study evaluated the effect of IR at 0.54 mGy/h and 10.9 mGy/h on 5.5 hpf embryos and showed that major developmental signalling pathways like Notch, RA signalling and apoptosis, were impaired during early zebrafish embryonic development^14^. However, this transcriptomics analysis was made after relative short irradiation time (5.5 hpf), and on developmental stage (mid-epiboly embryos) where the three embryonic layers (ectoderm, endoderm and mesoderm) are present but no cell-type specifications (such as neurogenesis or myogenesis) occurred yet. The developmental consequences of early perturbations in Notch or RA signalling on neurogenesis were thus not directly assessed in this study. Our analysis extend those findings in the field of chronic low doses and provides a deeper mechanistic understanding of the motility effects observed at the individual level. Our data provide evidence that both neuronal and muscle development are affected at stages where these processes are ongoing (24 hpf to 96 hpf). However, in contrast to Hurem *et al*.^14^, we did not detect drastic phenotypes such as embryonic deformities or mortality in zebrafish embryos exposed at dose rates as high as 50 mGy/h (total absorbed dose = 1.2 Gy at 24 hpf). Other studies that used either acute^35,36^ or chronic^13^ exposures to similar dose of IR did not detect strong developmental defects, in accordance with our data. Such discrepancy at the individual level could be due to differences in the maintenance of zebrafish embryos during the exposure to IR (for instance presence or absence of methylene blue in the embryos medium) or to the use of different gamma-sources (^60^Co and ^137^Cs).

Our transcriptomics analysis showed that only 5.6% of the DEG (78 genes) were common to all dose rates in 48 hpf larvae, while this number dropped to less than 1% at 24 hpf and at 96 hpf. We demonstrated that the criterion used to define DEG in our transcriptomics study (|fold change| ≥ 1.5 and adjusted p-value < 0.01) is robust, since we could validate down or up-regulation by qRT-PCR and RNA *in situ* hybridization. The GO term analysis of genes deregulated in common in all conditions at 48 hpf showed that neuronal development, apoptosis and steroid hormone signalling (which includes thyroid hormones, vitamin D, and RA^37^) were impacted at dose rate as low as 0.5 mGy/h.

The study of TF expression profiles is commonly used as a fingerprint of gene regulatory networks activity that can predict adverse outcomes observed at higher organisational levels. In our study the molecular profiling of TF highlighted similar molecular responses at 5 mGy/h and 50 mGy/h for all developmental stages analysed. In comparison the transcriptomics effects at 0.5 mGy/h were moderate, but neurogenesis effects were still detected at the molecular level. The proteomics analysis on 96 hpf larvae exposed to 5 mGy/h and 50 mGy/h confirmed our transcriptomics data, as neurogenesis, muscle development and RA signalling were also perturbed at the protein level. Perturbations in synapse organisation were highlighted in the proteomics and the transcriptomics data at 96 hpf and 48 hpf respectively. In addition, the TFBS analysis demonstrated the enrichment of MEF2 DNA binding sites in the promoter of the DEG of the 96 hpf larvae exposed at 50 mGy/h, a gene family is involved in the development of inhibitory and excitatory synapses in the brain of mouse embryos^38^. It is thus possible that synaptogenesis is impaired in the IR exposed 96 hpf zebrafish larvae. Caution must be taken to TF binding site prediction as it is prone to false positive discovery, but the high overlap (> 20%) between TF binding sites prediction and human ChIP-Seq data from the ENCODE project supports the notion that the transcriptional responses to IR could contain direct transcriptional targets of MEF2 or HES1.

RA signalling was perturbed at different levels in our global analysis. Indeed, the transcriptomics data showed a deregulation of the RA pathway at the gene level (Supplementary Fig. S8) and we found an enrichment of RARE in the promoters of DEG. In addition, vitamin A metabolic process, a synonymous term for the RA pathway, was also enriched significantly in the functional analysis of the proteomic data in 96 hpf larvae at 50 mGy/h and 5 mGy/h. These three pieces of evidences strongly suggest that RA signalling is impacted by chronic exposure to IR at 5 mGy/h and 50 mGy/h. RA signalling was also deregulated in the transcriptomics data gained at 0.5 mGy/h, but changes in gene expression were milder compared to the two highest dose rates. From these we can conclude that RA signalling, neurogenesis and somitogenesis are impaired in embryos exposed to dose rates higher than 5 mGy/h for a duration of at least 24 hpf. Lower dose rate (0.5 mGy/h) or shorter exposure time to IR led also probably to RA deregulation, as observed in our transcriptomics data at 24 hpf and as proposed in Hurem *et al*. on 5 hpf embryos^14^, but further functional analysis on RA signalling and its alteration by IR are needed to clearly establish a direct impact on neurogenesis and somitogenesis.

The RA pathway is a well-known morphogen involved in the anteroposterior patterning of the neural tube^39^ and its function is conserved among vertebrates. Another crucial role of RA is the control of primary neurons number in the spinal cord of fish and amphibians, which forms a neuronal circuit to coordinate escape movements and are thus crucial for larval survival after hatching^40–42^. The deregulation of the RA pathways could thus be one mechanism linking neurogenesis defects and the impairment of embryonic activities at 24 hpf and larval locomotion observed in our study. The RA pathway is also involved in somitogenesis^43^ and in neurites outgrowth^44^. Interestingly, evidences are also accumulating to link reduced or increased RA signalling with developmental defects like microcephaly and craniofacial malformations^45,46^. It is thus likely that the deregulation of this pathway can lead, at least in parts, to the congenital effects observed in exposed fetus in Chernobyl and Fukushima Daiichi. To investigate this possibility, it will be very interesting to raise the chronically irradiated zebrafish embryos to later developmental stages in order to study their brain size as well as the possible induction of apoptosis and DNA repair markers that could lead to cognitive dysfunctions and impairment of learning capabilities.

During neurogenesis, Notch-mediated lateral-inhibition plays a central role in the selection of undifferentiated progenitors to become committed neurons. In vertebrate embryos, the inhibition of the proneural genes *neurog1* and *ascl1*, which direct progenitors towards the neuronal fate, is mediated by transcription factors of the Hairy/Enhancer of Split family^47,48^. In addition, *her4* has another role during development as it is involved in the establishment of peripheral neuron projections in the trigeminal ganglion^49^. Our transcriptomics data showed that the *her4* family members (*her4.1, her4.2, her4.3* and *her4.4*) are downregulated (all fold change < −2 and adjusted p-value < 0.05) in 24 hpf embryos. By using whole mount *in situ* RNA hybridization, we found *her4.4* downregulation in groups of cells close to the central canal at all dose rates. The function of these cells is not known, but their location near the central canal suggests that they could be neuronal progenitors, and that IR could interfere with their differentiation into functional neurons. IR can also have an effect on neuronal migration. For instance, *in utero* acute irradiation of rat lead to ectopic neural populations in the cerebral cortex and hippocampus^50^. The disruption of expression pattern of *her4.4* observed in our study could thus be linked to an effect of IR on neuronal migration, but further studies (for instance by immunostaining of different neuronal populations by sox2, blbp and neuN) are required to better characterise this phenotype.

Interestingly, other members of the Hairy/Enhancer of Split family were, in contrast to the *her4* family, upregulated at 24 hpf after exposure to lR, for instance *her1* and *her7* (fold change < −2 and adjusted p-value < 0.05). These genes also function downstream of Notch-signalling but are necessary for the somite clock: the genetic network that regulates somite patterning in a time-dependant manner^51^. The analysis of *myogenin* (*myog*) expression pattern, a key TF during muscle development, highlighted clear ectopic expression in anterior parts of the trunk at 50 mGy/h, where *myog* should not be expressed anymore, irregular expression of *myog* at 0.5 mGy/h and 5 mGy/h. A significant overexpression of *myog* was detected in the mRNAseq data at 50 mGy/h, which is consistent with the supernumerary *myog* cells observed by RNA *in situ* hybridization. These observations on gene expression in the neural tube and somites are coherent with the impairments of the genetic networks regulating neurogenesis and muscle development observed at the molecular levels.

The integration of the multi-level data acquired in this study highlights that RA signalling is a major pathway perturbed during embryonic development after chronic exposure to IR. In the AOP framework we can thus propose that the disruption of RA signaling could be a major key event leading to the perturbation of somitogenesis and neurogenesis with harmful consequences on cognition and behavior.

## Material and Methods

### Animal experimentation and ethics

Animals were housed in the IRSN animal facilities accredited by the French Ministry of Agriculture for performing experiments on live zebrafish. Animal experiments were performed in compliance with French and European regulations on protection of animals used for scientific purposes (EC Directive 2010/63/EU and French Decret 2013–118). All experiments were approved by the Comité d’Ethique en Expérimentation Animale at the Institut de Radioprotection et de Sûreté Nucléaire (C2EA, IRSN, France) and authorised by the French Ministry of Research under the reference APAFIS#11488. Six to 9 month-old adults wild-type zebrafish (*Danio rerio*) of the AB strain were provided by Amagen (Gif-sur-Yvette, France). Fish were maintained under constant conditions in a ZebTEC system (Techniplast, France) at 28 ± 1 °C, 350–450 µS/cm, pH 7.5 and 12/12 h dark-light cycles. Fish were fed three times per day with TetraMin tropical fish food flakes (Tetra Werke, Germany). Health was monitored by daily inspection. Males and females were kept separated in 8 L tanks, 20 fish per tank, until mated. Males and females were kept separated in the same 1.7 L breeding tank (Beach Style Design, Techniplast, France) the night before mating, keeping the temperature constant at 28 ± 1 °C during the reproduction. Couples were mated the next morning during 15 min in fresh water. Eggs from all clutches were pooled and grown in 25 mL embryo medium (60 mg/L Instant Ocean, 0.01% (w/v) of methylene blue) under constant temperature (28.5 ± 0.2 °C) and dark-light cycles in MIR-154 incubator (Panasonic). Viable embryos were grown up to 120 hpf, reporting embryonic mortality daily. All experiments were conducted with a percentage of fertilization > 80%.

### Irradiation and dosimetry

Embryos were chronically exposed to gamma irradiation from the four-cell stage (1 hpf) up to 120 hpf by a ^137^Cs source of 370 GBq (Framatome ANP, Pierrelatte, France) in the MICADO experimental irradiation facility (IRSN, Cadarache, France). Clutches from 20 couples were polled and 50 fertilized embryos placed in 25 mL of 60 mg/L Instant Ocean, 0.01% (w/v) of methylene blue. Each condition consisted of three dishes (technical replicates), and the whole irradiation process was performed independently at least 3 times (three biological replicates). Non-fertilized eggs (coagulated eggs) were removed under the binocular at 24 hpf. Data loggers were placed in the MIR-154 incubators (Panasonic) in order to measure temperature stability during irradiation. Absorbed dose rates in 100 mm diameter Petri-dish containing 25 mL of fish water and air kerma rates were computed with the Monte-Carlo N-Particle transport code (MCNPX version X-24E). Operational dosimetry with radio-photoluminescent dosimeters (RPL, GD-301 type, Chiyoda Technol Corporation Japan) was used to confirm the simulations of the exposure to dose rates of 50 mGy/h, 5 mGy/h and 0.5 mGy/h and monitoring the radiation exposure. The effective dose rates were 46.80 ± 0.98 mGy/h, 5.13 ± 0.02 mGy/h and 0.48 ± 0.00 mGy/h (Supplementary Table T1). For hatching tests, homogeneous dose rate within 96 plates was checked, as before, for the three doses by MCNP computations followed by operational dosimetry with radio-photoluminescent dosimeters.

### Sequencing libraries preparation and data generation

Sequencing libraries were prepared from 1 µg of total RNA (extracted as described in Supplementary Material and Methods) with stranded TrueSeq mRNA version 2 kits and unique dual indexes from Illumina following manufacturer instructions. cDNA integrity, quality and concentration were assessed on DNA 1000 Chips (Bioanalyzer 2011, Agilent). Libraries were multiplexed at 2 nM and run on a NovaSeq. 6000 to produced 50 bases long paired-end reads at the Clinical Research Sequencing Platform (Broad Institute, MIT, United States).

### Transcriptomics data analysis

Quality over read length, duplication rates, insert size, adaptors contamination, nucleotide composition and mapping rates were controlled for each sample with an in house Perl script wrapping fastqc v0.11.5 (https://www.bioinformatics.babraham.ac.uk/projects/fastqc/), cutadapt v1.10^52^ and Picard v2.5.0 (http://broadinstitute.github.io/picard/). Raw reads were subjected to quality selection (Phred score Q > 30) and adapter trimming with Trimgalor v0.4.2. Between 77–132 millions of good quality reads were produced for each sample (Supplementary Table T2). Trimmed reads were mapped against the GRCz11 zebrafish reference genome using RNA-STAR v020201^53^, with the known exon-exon junctions from Ensembl release 95^54^. For all samples, the percentage of uniquely mapped reads against the reference genome was > 70%, aligned pairs duplication level < 0.7% and percentage of adapters < 0.1%. Details on Phred quality over read length, mapping statistics against the zebrafish genome and %GC are provided in Supplementary Fig. S2. Hierarchical clustering of the variance stabilized expression data was produced with Pearson’s correlation and complete-linkage method. Potential outliers were removed in order to increase the power of the differential analysis. This led to the selection of 46 samples from the original set of 53 samples (Supplementary Fig. S1). Transcriptomics data were analysed in R using rlog normalised expression data and differential expression analysis obtained with DESeq. 2 v1.22.2^55^. Genes with |fold change| ≥ 1.5 and adjusted p-value < 0.01 (False Discovery Rate) were considered as differentially expressed. Hierarchical clustering of normalised expression data (rlog) or fold changes from DESeq. 2 were made with the R package *hclust* using Pearson’s correlation and the complete-linkage method.

### Functional enrichments of transcriptomics data

Gene ontology (GO)^56^ and KEGG^57^ functional enrichments were performed using the R packages *topGO*^58^ and *clusterProfiler*^59^. Enrichments with GO were performed with the zebrafish deregulated genes obtained from DESeq. 2. For KEGG analysis, we used the human gene orthologues with at least 30% homology using *biomaRt*^60^. Enrichments with p-value from Fisher’s exact test < 0.01 were considered significant. MA-plots, heat maps, histograms and Venn diagrams were produced using the R package *ggplot2*^61^. Selection of genes involved in the RA pathway were fetched with *biomaRt* using the GO terms: retinol metabolic process (GO:0042572) and retinoid metabolic process (GO:0001523). The selection of genes involved in neurogenesis was performed the same way using the GO term: neurogenesis (GO:0022008). Selection of DNA-binding TF was made, as published before^30^, using a selection of 609 curated DNA-binding domains, except that the list was updated for Interpro v72^62^ (Supplementary Table T3). Ensembl genes identifiers for all TF mapped in GRCz11 release 95 were retrieved using *biomaRt*. A total of 462 unique TF with a significant change (|fold change| ≥ 1.5 and adjusted p-value < 0.01) in at least one comparative analysis were used for hierarchical clustering of averaged fold changes.

### Transcription factor binding site analysis

Significantly (|fold change| ≥ 1.5 and adjusted p-value < 0.01) up and downregulated genes after exposure to 50 mGy/h at 24 hpf, 48 hpf and 96 hpf were searched for enrichments of TF DNA binding sites in their promoter sequences. A set of 5000 randomly chosen promoters was used as background. Promoter sequences were defined as DNA genomic sequences 2 kb upstream and 50 bp downstream the transcriptional start site and were retrieved via the Perl Applied Program Interface from Ensembl release 95. The resulting promoter sequences were searched for enrichment of TFBS with OPOSSUM v3^63^ and hidden Markov matrix models from Jaspar core-vertebrate database v2018^64^. Binding sites with Z-score > 6 and Fisher-score > 3 were considered significantly enriched. ChiP-Seq data in Bed format were retrieved from ENCODE^31^ for HES1 (ENCSR091JXL) and MEF2B (ENCSR177VFS). Enriched TFBS from OPPOSOM and binding sites from ChIP-Seq data were compared at the level of genomic coordinates by fetching human orthologues of zebrafish genes and checking overlap in 2 kb promoters of each potential target gene using the R package *GenomicRanges*^65^.

### Mass spectrometry analysis

Mass spectrometry analysis were done as already described previously^66^. Briefly, peptides obtained after trypsinisation were diluted in solvent A (0.1% v/v formic acid in 2% v/v acetonitrile) and concentrated using a pre-column (C18 PepMap100, 2 cm × 100 µm I.D, 100 Å pore size, 5 µm particle size, Dionex). Peptides were separated on a reverse phase Liquid Chromatography column (PepMap RSLC C18, 50 cm × 75 µm I.D, 100 Å pore size, 2 µm particle size, Dionex). Peptides were eluted by a two steps linear gradient of 4–20% v/v acetonitrile/H_2_O, 0.1% v/v formic acid for 220 min and 20–45% v/v acetonitrile/H_2_O, 0.1% v/v formic acid for 20 min at 300 nL/min flow rate and 40 °C. Peptide ionization was performed using an EASY-Spray nanosource (2.2 kV and capillary temperature set at 275 °C), and MS spectra acquired with an Orbitrap Fusion Lumos Tribrid Mass spectrometer (Thermo Fisher Scientific) using the following settings: m/z 400–160, FWHM resolution of 120 000 (measured at 200 m/z), AGC target set at 4.105 with 50 ms Maximum Injection Time, time between Masters Scans set to 3 seconds. The more abundant precursor ions were selected for MS/MS analysis. Collision induced dissociation fragmentations at 35% were performed and analyzed in the ion trap using: “Inject Ions for All Available Parallelizable time”, maximum injection time of 300 ms, AGC target set at 4.10^3^, charge state screening enabled to include precursors with 2 and 7 charges, dynamic exclusion enabled with one repeat count and duration of 60 sec.

### Quantitative proteomics processing

Data processing was performed using MaxQuant v1.6.1.0, computing relative intensities based on label-free quantification (LFQ) with the MaxLFQ algorithm. Mass spectra and differential protein expression analysis were performed as described before using Perseus v1.6.1.3^66^. Briefly precursors ions identification was performed using the *Danio rerio* UniProt protein database (12^th^ of February 2019 with 58994 entries) supplemented with 245 proteins commonly found as contaminants and a false positive rate of 1% for protein identification. Normalised intensity LFQ were log2 transformed to obtain a normal distribution and differentially expressed proteins identified by a multiple ANOVA t-test or student t-test, controlling the false positive rate (1% with 250 permutations). |fold-change| ≥ 1.5 and p-values < 0.01 were considered as significant. Functional analysis of proteomic data was performed with topGO using zebrafish proteins identifiers.

### γ-H2AX analyses

Dechorionated 24 hpf embryos and 48 hpf larvae were fixed in 4% w/v paraformaldehyde (PAF) overnight at 4 °C and transferred in 100% v/v methanol at −20 °C until use. Samples were rehydrated in a decreasing gradient of methanol diluted in phosphate-buffered solution (PBS) v/v at 70%, 50% and 25% for 5 min each and washed 4 times 5 min in PBS +0.1% v/v Tween 20 (PBST). Embryos and larvae were permeabilized with proteinase K at 10 µg/mL for 3 and 30 min respectively, and fixed for 20 min in 4% w/v PAF at 4 °C. The samples were washed 5 times 5 min in PBST, incubated 3 hours at room temperature with blocking buffer 0.2% w/v BSA and 1% v/v DMSO diluted in PBST, and incubated at 4 °C overnight with the γ-H2AX antibody (GTX127342, GeneTex, France) diluted at 1:200 in blocking solution. The next day, samples were washed five times 15 min in PBST, and incubated 2 h at room temperature with anti-rabbit-FITC antibody (F0382, Sigma-Aldrich, France) diluted to 1:200 in PBST. After 5 washes of 5 min in PBST, nuclei were stained 1.5 h in 300 nM DAPI in PBS, washed 2 times 5 min in PBST and kept protected from light at 4 °C until confocal analysis. Image acquisition was performed using a laser scanning confocal microscope (Carl Zeiss, France) equipped with a 20x dry objective. For FITC fluorescence, excitation was set to 488 nm and emission acquired between 510/550 nm. For DAPI detection, excitation was performed at 405 nm and detection between 420/500 nm. Embryos and larvae were mounted on 35 mm imaging dish with a polymer coverslip bottom (Ibidi, Germany) and oriented laterally in a 2% w/v methyl-cellulose solution for image acquisition. Ten embryos or larvae per condition were analysed, focusing in the trunk region located above the yolk extension. Smooth filtering was applied on TIFF images in ImageJ^67^ to increase the signal to noise ratio and background subtracted for each image. An average of 50 regions of interest (ROI) were chosen per embryos or larvae in duplicated experiments. At least 1200 nuclei were detected for each condition with the ImageJ tool ROI manager, and γ-H2Ax foci counted with the ImageJ function *Find Maxima* to compute the number of positive foci per nuclei. Analyse of normality showed that these data were not normally distributed. Hence, a Kruskall-wallis test was performed followed by pairwise Wilcoxon rank sum test as post-hoc tests and p-values were adjusted by the Holm method. Adjusted p-values < 0.05 were considered as significant.

### Body length measurement

Ten 48 hpf old larvae were immobilized in 2% w/v methylcellulose with 0.02% w/v MS-222 (Sigma-Aldrich, France) in PBS 1X to avoid movement during measurement. Pictures were taken under a Nikon SMZ800 stereomicroscope equipped with a high-resolution camera (acA1300-50gm, Basler, Germany). Images analysis to measure body length were performed with DanioScope v1.1.110 (Noldus Information Technology, Netherlands) from 67 to 101 larvae per condition. After control of normality, a one way ANOVA test was performed.

### Cardiac activities

Larvae were acclimated for 30 min in the illuminated incubator (AquaLytic, Germany) at 28 ± 1 °C located in the behavioural room. Ten 48 hpf larvae immobilized in 2% w/v methylcellulose with 0.02% w/v MS-222 (Sigma-Aldrich, France) in PBS 1X to avoid movements with an acclimation step of two minutes in the behavioural room. MS-222 concentration used in the present study was about 2 times higher than the EC_50_ found for 72 hpf zebrafish larvae 1 h after exposure^68^, in order to ensure that larvae were immotile during the short time of video recording (< 3 min). All larvae swam normally when placed back in clean embryo medium. Video recording was performed under a stereomicroscope (Nikon SMZ800) connected to a high-resolution camera (acA1300-60gm, Basler, Germany). A delay of 30 sec was respected before each video acquisition to avoid stress bias. The Media Recorder Software v.4.0.542.1 (Noldus Information Technology, Netherlands) was used for recording 30 sec long videos at frame rate = 25 Hz. Analysis were performed using DanioScope Software v.1.1.110 (Noldus Information Technology, Netherlands). The cardiac frequency measurement was computed from at least 61 larvae per condition. As these data were not normally distributed, a Kruskall-wallis test was performed followed by pairwise Wilcoxon rank sum test as post-hoc tests and the p-values were adjusted by the Holm method. Adjusted p-values < 0.05 were considered significant.

### Embryonic activity and larval motility tests

For all behavioural experiments embryos or larvae were acclimated for 30 min in the illuminated incubator (AquaLytic, Germany) at 28 ± 1 °C located in the behavioural room (itself set to 28 °C). For the embryonic activity at 24 hpf, ten embryos were immobilized in 2% w/v methylcellulose in PBS to avoid drift during video recording performed under a stereomicroscope (Nikon SMZ800) connected to a high resolution camera (acA1300-60gm, Basler, Germany). A delay of 2 min was respected before each video acquisition to avoid stress bias. Media Recorder Software v.4.0.542.1 (Noldus Information Technology, Netherlands) was used for recording 5 min long videos at frame rate = 25 Hz. Analysis were performed using DanioScope Software v.1.1.110 (Noldus Information Technology, Netherlands). Burst activity, determined as the percentage of time the embryo is moving, was computed from 147 to 250 embryos per condition in triplicates.

For the visual motor test, triplicated experiments of 56 to 128 larvae (120 hpf) per condition were placed in 24 wells plates filled with 2 mL of embryo medium. Test was performed the afternoon which is the most stable period for larval activity^69^. Control and exposed larvae were placed within each plate to avoid batch effects and analysed in the DanioVision Observation Chamber coupled with the DanioVision Temperature Control Unit (Noldus Information Technology, Netherlands) set to 28 ± 0.1 °C. An acclimation period of 10 min with light off was allowed for each plate inside the DanioVision Observation Chamber. Recording of videos started to track larval motility (defined as the distance moved in mm) over 3 phases: i) 5 min with light on, ii) 5 min light off and iii) 5 min light on. Videos were recorded and analysed with Ethovision XT Software v.13.0.1216 (Noldus Information Technology, Netherland). Statistical analysis of embryonic and larval locomotor activities was made by applying a linear mixed effect model with treatments as fixed effect using the R package *nlme*. Indeed, larvae within one replicate are more similar to each other than larvae in other replicates, especially in terms of growth since the videos were not strictly taken at the same time during the day. Replicates were thus used as a random effect in the model. Permutation tests, based on the linear mixed effect model were used to compare each treatment to control. Permutation tests were made with the R package *pgirmess*. p-value < 0.05 were considered as significant.

### Transmission electron microscopic analysis

Samples preparation and TEM microscopy analysis were performed as described previously^70^. More specifically, 96 hpf larvae were individually fixed with 2.5% w/v glutaraldehyde in 0.1 M, pH 7.4 sodium cacodylate buffer for two days at 4 °C. Larvae were washed three times 5 min with the same buffer and post-fixed 1 h with 1% w/v osmium tetroxide in cacodylate buffer. Embryos were dehydrated in increasing concentrations of ethanol and embedded in monomeric resin (Epon 812, Electron Microscopy Sciences, Hatfield, USA). Sections for optical and electron microscopy of 500 nm and 80 nm respectively were obtained using an ultramicrotome UCT (Leica Microsystems GmbH, Wetzlar, Germany). TEM observations were made on 80 nm thick and 3 mm long sections mounted on copper grids. Images were taken with an electron microscope (Tecnai 12G Biotowin, Netherlands) equipped with a CCD camera (Megaview III, Olympus Soft Imaging Solutions GmbH, Germany) with an accelerating voltage of 100 KeV. At least 20 ROI were taken for each individual in triplicates. ROI were categorised for the two highest dose rates (50 mGy/h and 5 mGy/h) and control groups according to the degree of surface damage observed: no damage, moderate or extensive damage. The percentage of TEM images defined as ROI was computed from at least 21 independent ROI in triplicate (n > 71 for each group). Data being categorical, the statistical analysis was done using a Pearson Chi-squared test and the p-values adjusted by the Holm method. Adjusted p-value < 0.05 were considered as significant.

### Whole mount RNA *in situ* hybridization and image acquisition

Antisense RNA probes labelled with digoxygenin (DIG) for *her4.4* and *myogenin* were generated from 24 hpf embryos as follows. Total RNA extraction from 24 hpf embryos was performed using a TRIzol/chloroform extraction (Life Technologies), with the exception that an additional chloroform extraction step was performed before the isopropyl alcohol precipitation of nucleic acids. Reverse transcription was made using SuperScript III (Life Technologies), dNTP, polydT primers and 1 µg of total RNA following manufacturer instructions. Gene specific PCR amplification was done using Taq platinum kit (Life Technologies) with 1 µL of RT and using the primers pairs: *her4.4* forward CCTGACGGAGAACTGAACACA, *her4.4* reverse GCAGAGCAAGAATCCTTCAATGA, *myogenin* forward AGTTGGTGTGGAGCAGTTGT, *myogenin* reverse GCCTTCCTGACTGCCTTAAGT. PCR were validated by electrophoresis on 1.2% w/v agarose gel and cloned into PCRII dual promoter bacterial vector following kit instructions (TOPO TA Cloning, Invitrogen). Chemically competent TOP10′ *E. coli* (Invitrogen) were transformed with 2 µL of ligation product, plated on Petri dish with LB agar with 100 µg/mL Ampicillin and incubated over night at 37 °C. At least three colonies for each construct were picked and grown individually in liquid LB medium with 100 µg/mL of Ampicillin overnight at 37 °C. Plasmid DNA were extracted following the alkaline lysis protocol and sent for Sanger sequencing to check cDNA identity and orientation of cloning in the PCRII vector. Antisense RNA probes were generated by cutting the DNA plasmid at the 5′ end of the cDNA, followed by a phenol/chloroform extraction of the linearized vector, and *in vitro* transcription of the Dig RNA probes with T7 or Sp6 RNA polymerase and the Dig-UTP labelling mix (Roche). Whole mount RNA *in situ* hybridization labelling was made following the protocol from Zfin (https://zfin.org/ZFIN/Methods/ThisseProtocol.html). At least 10 embryos were used per condition. Briefly, 24 hpf embryo were fixed overnight at 4 °C in 4% w/v PAF and transferred in 100% v/v methanol at −20 °C until use. Samples were rehydrated in a decreasing gradient of methanol diluted in PBS v/v at 70%, 50% and 25% for 5 min each and then washed 4 times 5 min in PBST. Embryos and larvae were incubated in proteinase K at 10 µg/mL for 3 min, fixed for 20 min in 4% w/v PAF at 4 °C and washed 3 times 5 min in PBST. Embryos were incubated 4 hours at 65 °C with RNA hybridization buffer and put in contact overnight with 100 ng of gene specific DIG-labelled RNA probes diluted in RNA hybridization buffer. Embryos were washed 15 min at 65 °C in four consecutive washing buffers, 3 times in PBST at room temperature, incubated 3 h at room temperature with blocking buffer and incubated at 4 °C with anti-Digoxigenin-AP Fab fragment antibody (Roche Diagnostics GmbH, Germany) diluted 1:4000 in blocking buffer. Then, embryos were washed 5 times with PTW buffer before staining with NBT/BCIP (Roche Diagnostics GmbH, Germany). Each *in situ* hybridization staining was repeated at least 2 times. Expression patterns were observed under a Nikon binocular (SMZ800N) equipped with a Toupcam U3CMOS 10000KPA camera (ToupTek Photonics, China). Images of representative embryos were taken for each group, when phenotypes could be observed under the binocular in at least 50% of the embryos (n = 10).

### Supplementary material and methods

A description of protocols for hatching rate, extraction of total RNA and proteins, as well as Taq-man assay can be found in Supplementary Material and Methods.

## Supplementary information

Supplementary Information

Supplementary Table T3

Supplementary Table T4

Supplementary Table T5

## Data Availability

All transcriptomics data are accessible in the GEO repository under the accession number GSE134634.
