# Supplementary material for: A systems biology approach reveals neuronal and muscle developmental defects after chronic exposure to ionising radiation in zebrafish
Source: Sci Rep. 2019 Dec 27;9:20241. doi: 10.1038/s41598-019-56590-w (PMC6934629; doi:10.1038/s41598-019-56590-w)
Supplement: Supplementary file 1 — Supplementary Information [file 41598_2019_56590_MOESM1_ESM.pdf]

A systems biology approach reveals neuronal and muscle developmental defects after chronic exposure to ionising radiation in zebrafish

Sophia Murat El Houdigui[a], Christelle Adam-Guillermin[c], Giovanna Loro[a], Caroline Arcanjo[a], Sandrine Frelon[a], Magali Floriani[a], Nicolas Dubourg[a], Emilie Baudelet[b], Stéphane Audebert[b], Luc Camoin[b] & Olivier Armant[a*]

Supplementary Information

Supplementary Table T1: Results of MCNPX simulations and operational dosimetry obtained with radio photoluminescent dosimeter (mGy/h) at the target dose rates of 50 mGy/h, 5 mGy/h and 0.5 mGy/h. The corrected dose rate takes under account the layer of medium in the petri dish, considering that embryos younger than 48 hpf are located at the bottom and swimming larvae (> 48 hpf) 1 mm below water level. Tests 1 to 6 correspond to simulations and measures for 24 hpf embryos in the chorion at the bottom of the petri-dish, while tests 7 to 12 correspond to dose corrected for 48 hpf swimming larvae. The average and standard deviation (sd) are indicated for each condition.

Supplementary Table T2: Quality control of sequencing reads of the 58 samples analysed by mRNAseq. Replicates number, dose rate, irradiation batch and developmental stage are indicated for each sample, as well as the reads number obtained and the % of reads with Phred score (Q) > 30 (R1: read1, R2: read2).

Supplementary Table T3: List of Interpro domains specific to transcription factors. Functional category “a” indicates the high probability of the Interpro domain to be part of genuine transcription factors, as described before [30] (based on Interpro v25). The last column indicates if the domain is novel (present in the Interpro v72 database) or deprecated (absent in Interpro v72).

Supplementary Table T4: List of significantly (|fold change| ≥ 1.5 and adjusted p-value ≤ 0.01) deregulated zebrafish genes in each pair-wise comparative analysis.

Supplementary Table T5: List of zebrafish GO terms enriched in each comparative analysis. P-values from Fisher’s exact test are indicated.

Supplementary Table T6: List of zebrafish GO terms enriched in the 78 genes mis-regulated in common at the three dose rates at 48 hpf. P-values from Fisher's exact test are indicated.

Supplementary Table T7: List of the 56 zebrafish proteins differentially expressed in 96 hpf larvae exposed at 5 mGy/h.

Supplementary Table T8: List of the 88 zebrafish proteins differentially expressed in 96 hpf larvae exposed at 50 mGy/h.

Supplementary Figure S1: Assessment of biological variability of samples by hierarchical clustering. (**a**) Hierarchical clustering of all biological replicates. Potential outliers are indicated in red. (**b**) Hierarchical clustering of biological replicates after removal of potential outliers. The irradiation batch is indicated for each sample (I10, I17, I32), as well as the developmental time (24 hpf, 48 hpf and 96 hpf), the exposed condition (C: control, 05mG: 0.5 mGy/h, 5mG: 5 mGy/h, 50mG: 50 mGy/h) and the replicate number. Yellow indicates small and red high Euclidean distances.

Supplementary Figure S2: Quality controls of sequencing data. (**a**) Mean Phred Score quality (Q) across each base in the reads for 53 samples. (**b**) (%) GC content of reads for 53 samples (as read counts). (**c**) % of mapped reads falling into genomic features.

Supplementary Figure S3: Correlation of log2(fold change) obtained by TaqMan quantitative RT-PCR and mRNAseq. Spearman correlation coefficient (rho) are indicated.

Supplementary Figure S4: Dot plot of zebrafish GO terms. Top 10 pathways obtained from GO enrichment sorted by significance based on p-values from the Fisher's exact test.

Supplementary Figure S5: Dot plot of GO pathways using human orthologues. GO terms are sorted by significance based on p-values from the Fisher's exact test.

Supplementary Figure S6: Mean of normalised expression, with standard error bars, over developmental time at the three dose rates and in control condition. *: significant change (|fold change| ≥ 1.5 and adjusted p-value ≤ 0.01).

Supplementary Figure S7: Volcano plots of the 1084 genes involved in neurogenesis (annotated in the Gene Ontology database with the term GO:0022008). Each dot

corresponds to a gene. Red: significant differential expression (|fold Change| ≥ 1.5 and adjusted p-value ≤ 0.01), grey: no change.

Supplementary Figure S8: Heatmap of the average of log2 fold change (from mRNAseq) of 12 genes expressed consistently in all conditions and involved in the RA pathway (annotated with the GO terms GO:0042572, retinol metabolic process, or GO:0001523, retinoid metabolic process). Red color indicates upregulation and blue downregulation compared to controls (white no change). 0.5: 0.5 mGy/h, 5: 5 mGy/h, 50: 50 mGy/h.

Supplementary Material & Methods

Analysis of hatching rate

160 fertilized eggs per condition were distributed individually into 96 wells plates containing 250 µL of zebrafish embryo medium. Embryonic development was observed daily under the binocular (Leica EZ4HD, France). The number of hatched eggs was recorded at 53, 55 and 72 hpf. The hatching rate was determined as a proportion of hatched eggs over the total living eggs at a given time.

RNA extraction and TLDA TaqMan array

Biological replicates (3 to 6 per condition) were made from pools of 40 embryos or larvae at 24 hpf, 48 hpf and 96 hpf. Total RNA extraction was performed using extraction by grinding (Precellys 24, Bertin Instruments, France) with ceramic beads tubes (CK14 tubes, Bertin Instruments, France) and a TRIzol/chloroform extraction (Life Technologies). RNA integrity (RIN), quality and concentration were assessed using RNA Nano Chips (Bioanalyser 2011, Agilent). No signs of RNA degradation were detected, all samples had a RIN > 8. Reverse transcription was made from 0.1 µg of total RNA with the High Capacity cDNA Reversion Transcription Kit (Applied Biosystems) following manufacturer instructions. Quantitative PCR were made on a QuantStudio 7 Flex Real-Time PCR system (Applied Biosystems) with 384-well microfluidic TaqMan Array Card (Applied Biosystems). Comparative threshold (Ct) were analysed by ExpressionSuite Software for QuantStudio 6 & 7 Flex Real Time PCR System (Applied Biosystems) and exported to DataAssist v3.01 (Applied Biosystems). Five

endogenous controls (*apoa1a*, *cdk4*, *cox6a1*, *pcna* and *psmb5*) were used to normalize expression. Fold change were calculated compared to control group. Pearson coefficient was calculated between TaqMan Array Card fold change and RNAseq data fold change with R software.

Protein extraction

Proteins were extracted from 96 hpf larvae (n=20) in triplicated experiments in RIPA buffer supplemented extemporaneously with 1 mg/mL leupeptin, 1 mg/mL aprotinin, 1 mM phenylmethylsulfonyl fluoride (Sigma, France). Samples were homogenized twice at 5000 rpm for 20 sec in the Precellys grinder system (Bertin Technologies, France) at 4 °C. After 15 min incubation on ice, lysates were centrifuged at 5000 g at 4 °C for 10 min and supernatant used for protein quantification with BCA kit (Thermo Scientific) according to the manufacturer's instructions. 20 µg of proteins were precipitated on ice for 20 min by the addition of 10 % v/v TCA (Sigma, France). Pellets were washed with ice-cold ethylacetate/ethanol (v/v), and resuspended at 30 °C in the dark under agitation at 1600 rpm in UTC924 buffer (9 M urea, 2 M thiourea, 4% (w/v) CHAPS, 20 mM Tris, pH 9.5) for 1.5 hours. The pH was adjusted to 8.5-8.6 with 50 mM Tris HCl pH 8.5 to get a final concentration of 2-3 µg/µL of proteins. Electrophoresis of 15 µg of proteins per replicate were done on a NuPAGE gel 4-12% (Life Technologies) for 6 min at 80 V using in MOPS buffer (Thermo Fisher Scientific) and stained by coomassie blue (Imperial protein stain, Thermo Fisher Scientific). Protein bands were then excised with a scalpel and digested overnight at 37°C with 0.6 µg trypsin (Promega, USA).

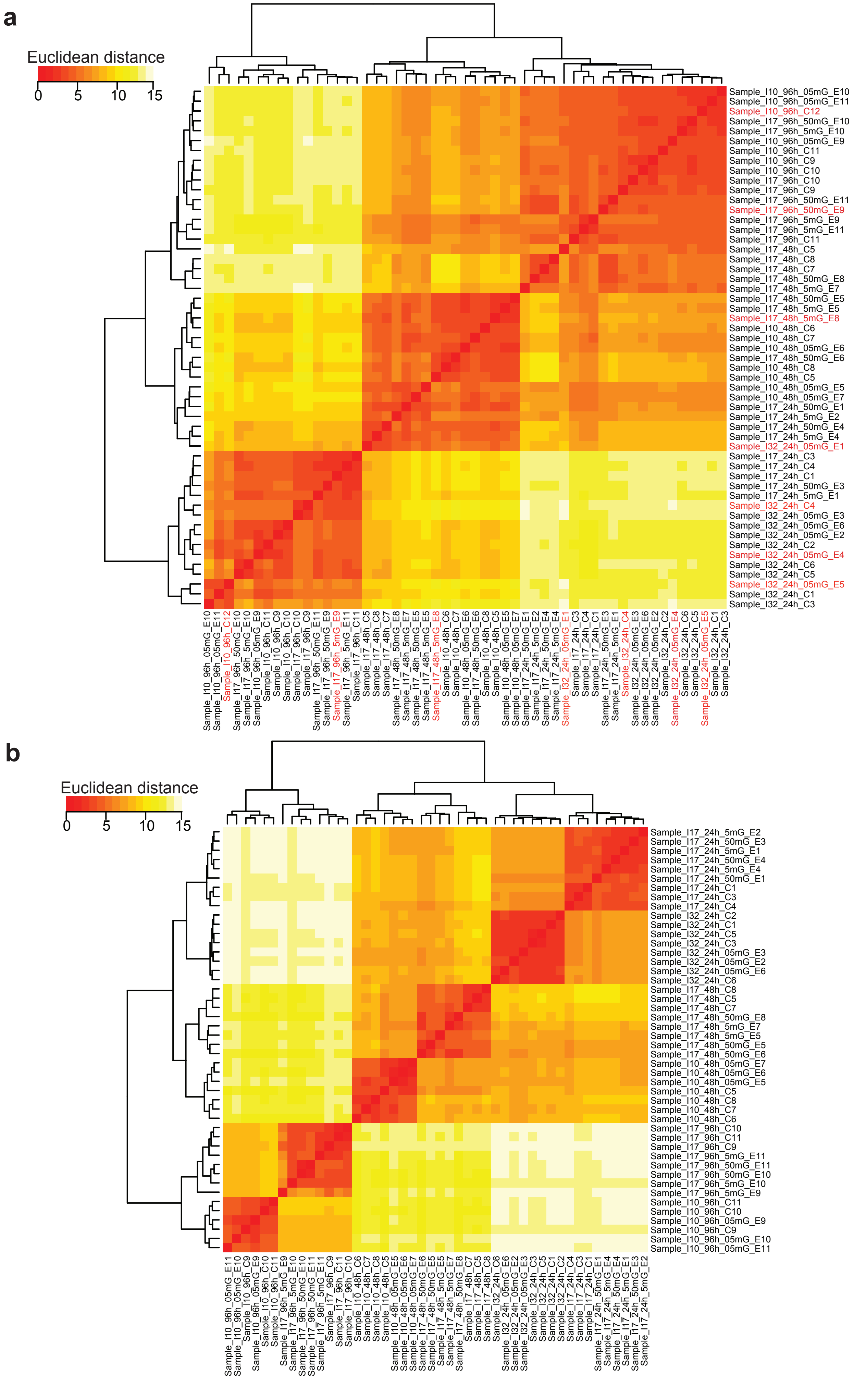

Supplementary Figure S1

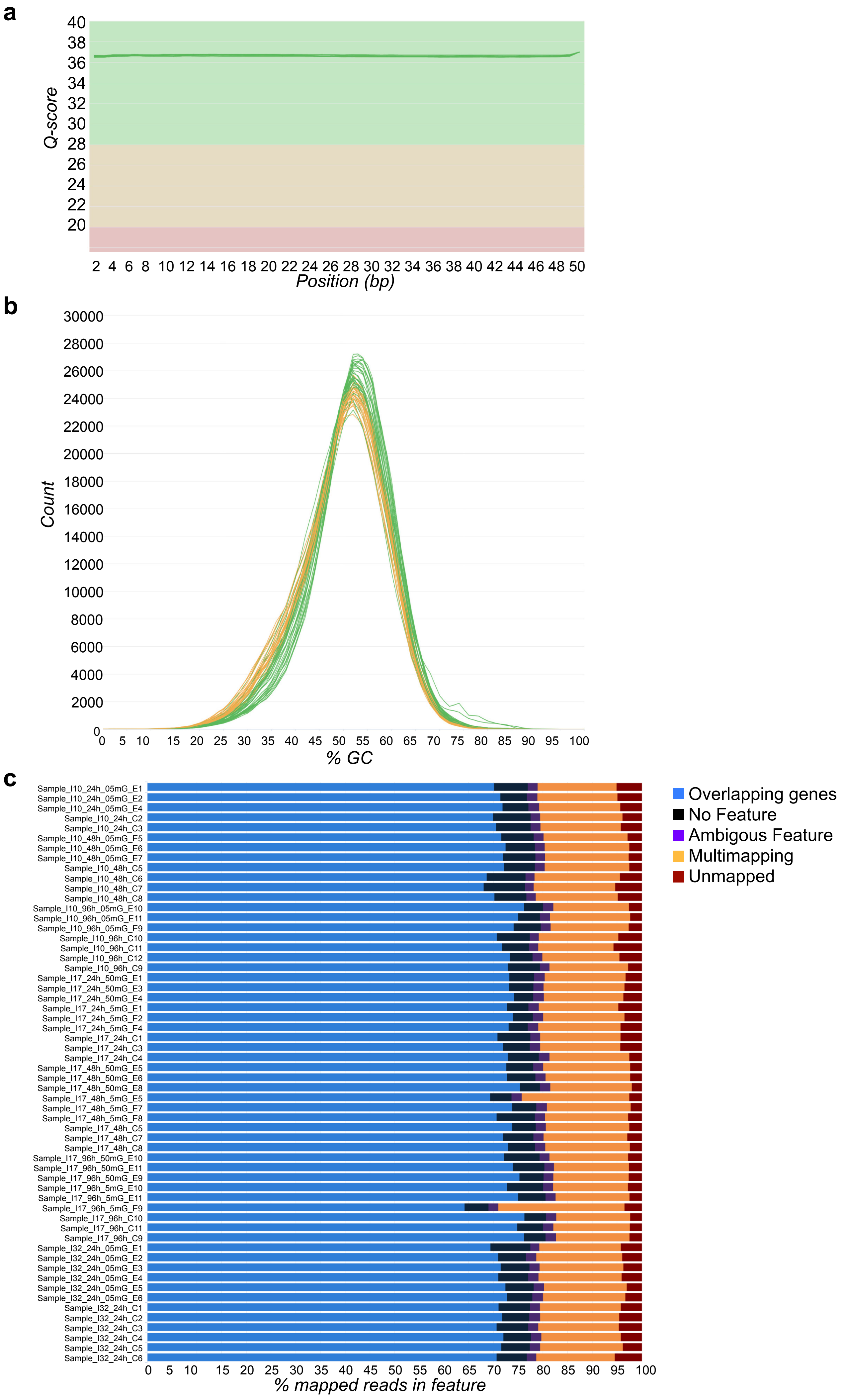

Supplementary Figure S2

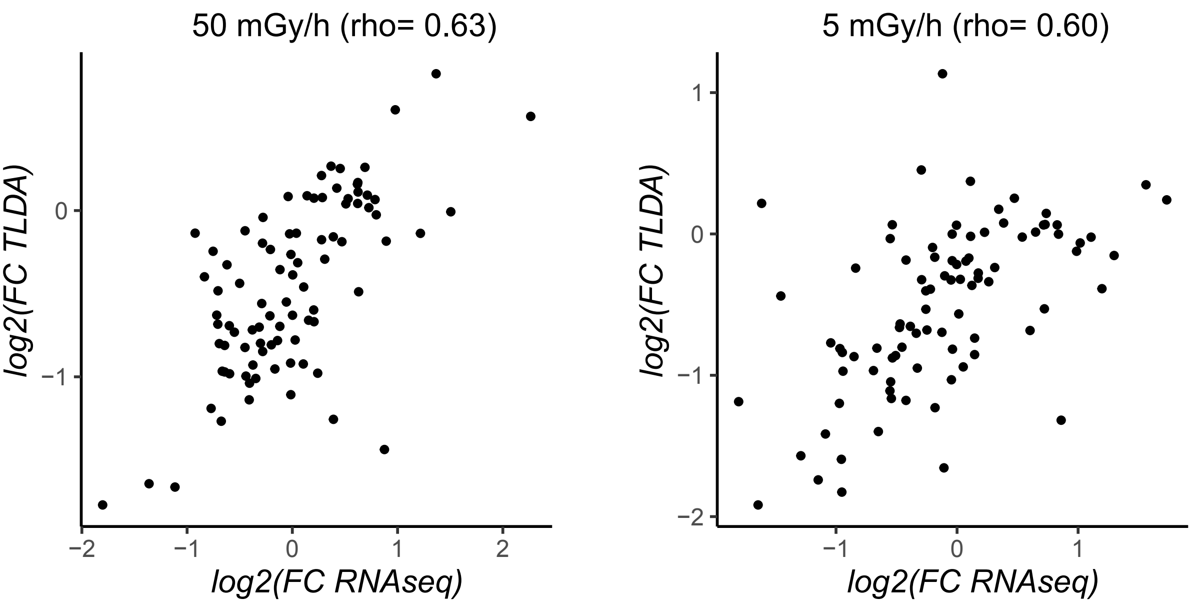

Supplementary Figure S3

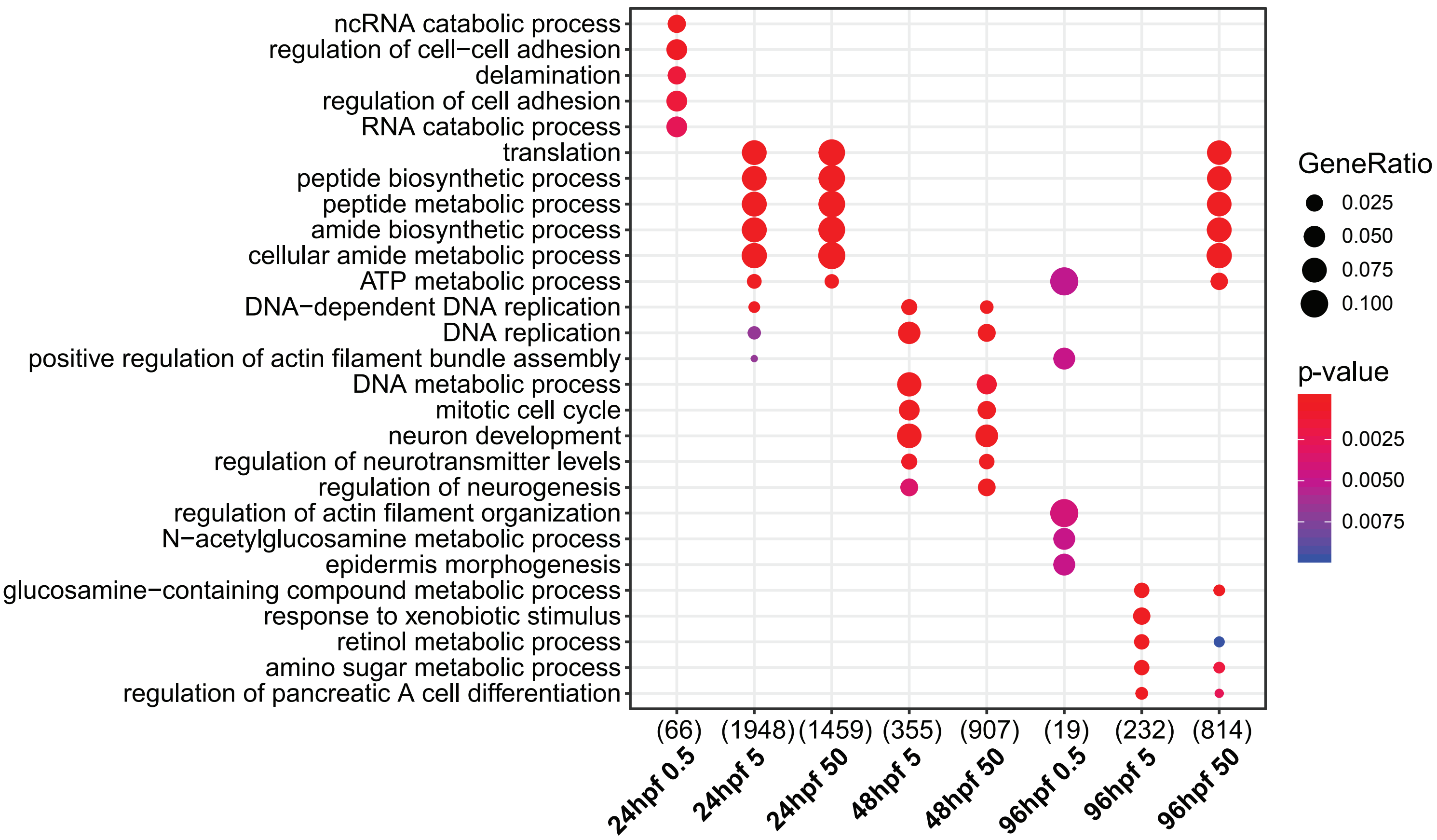

Supplementary Figure S4

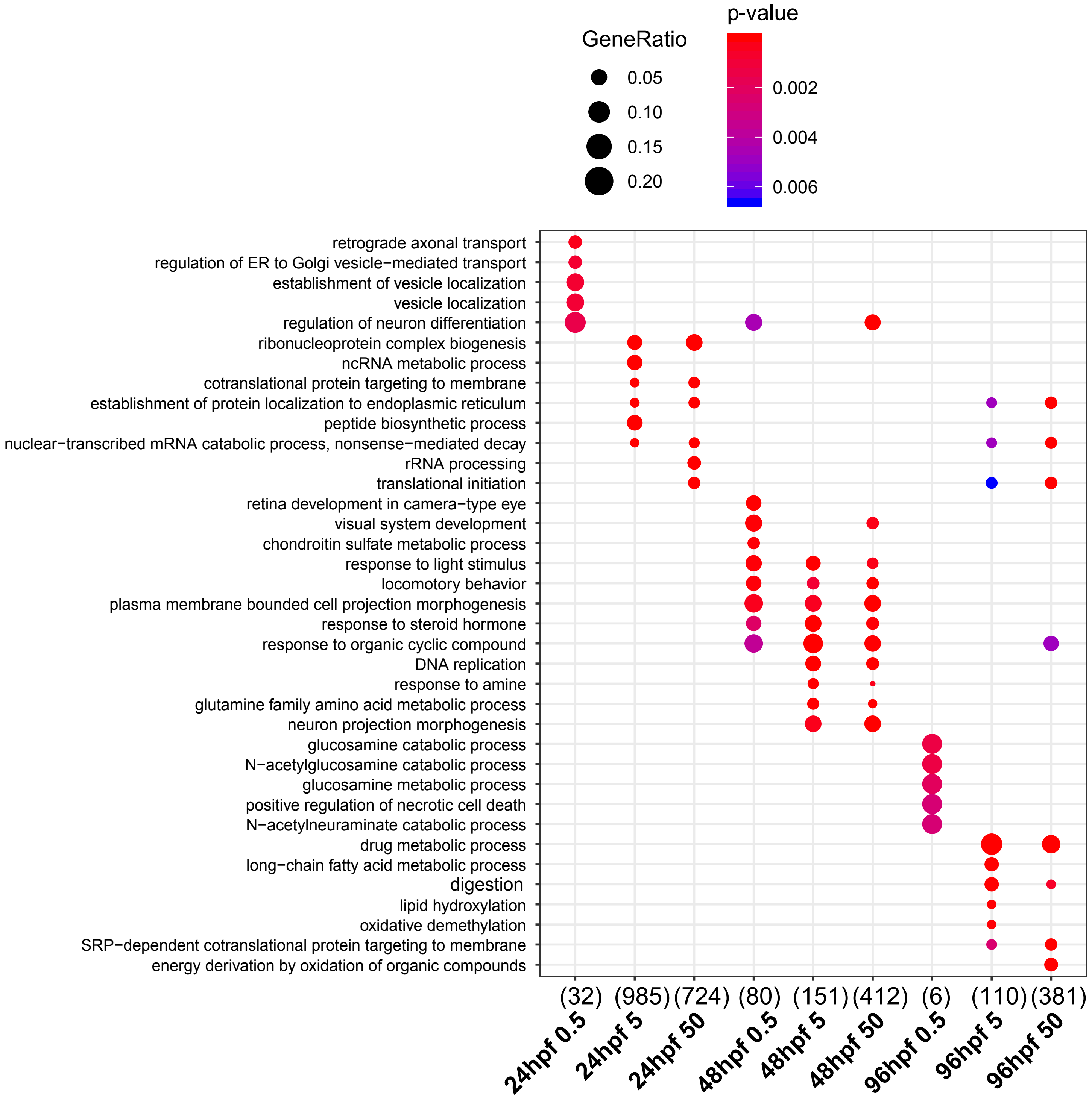

Supplementary Figure S5

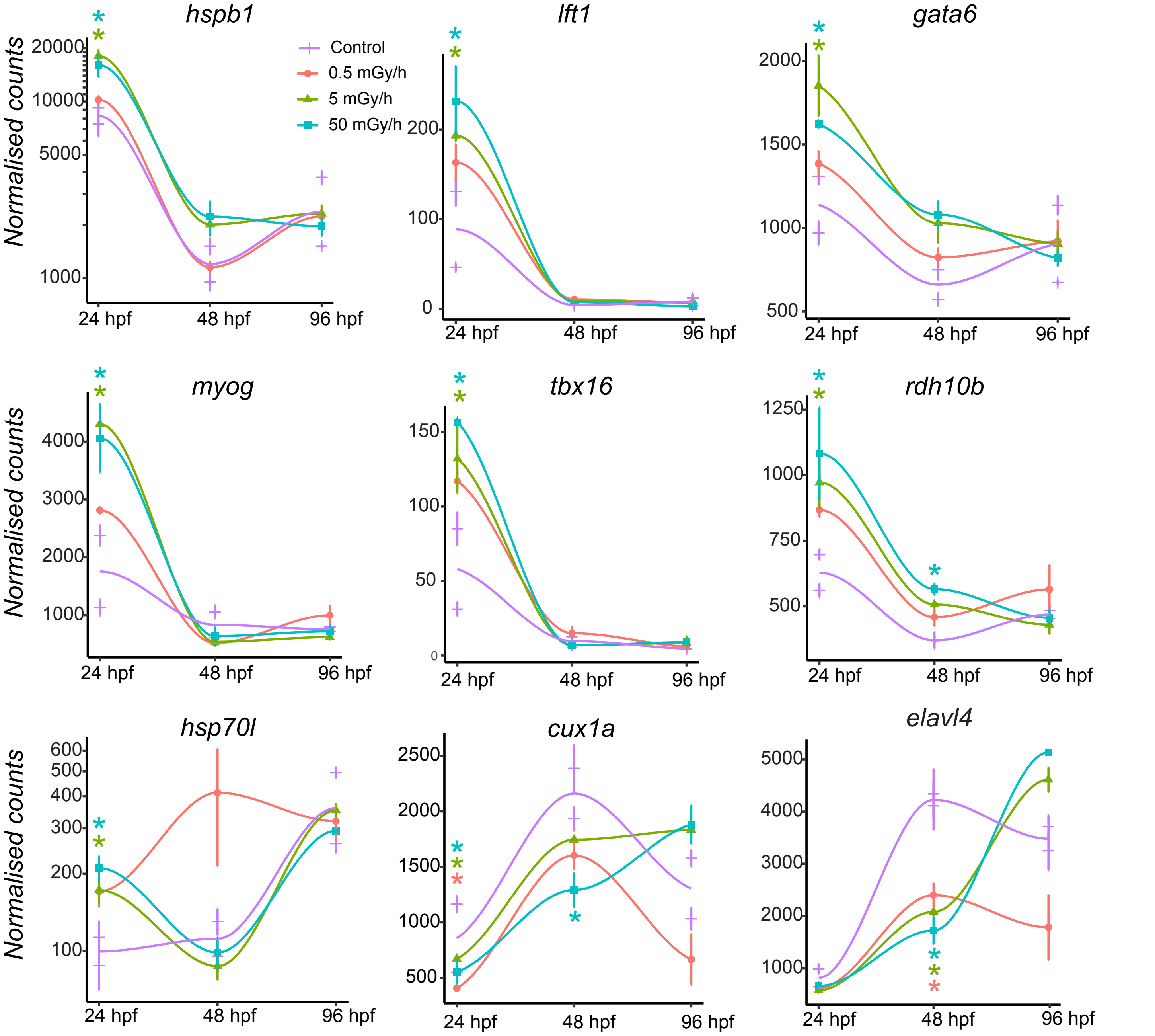

Supplementary Figure S6

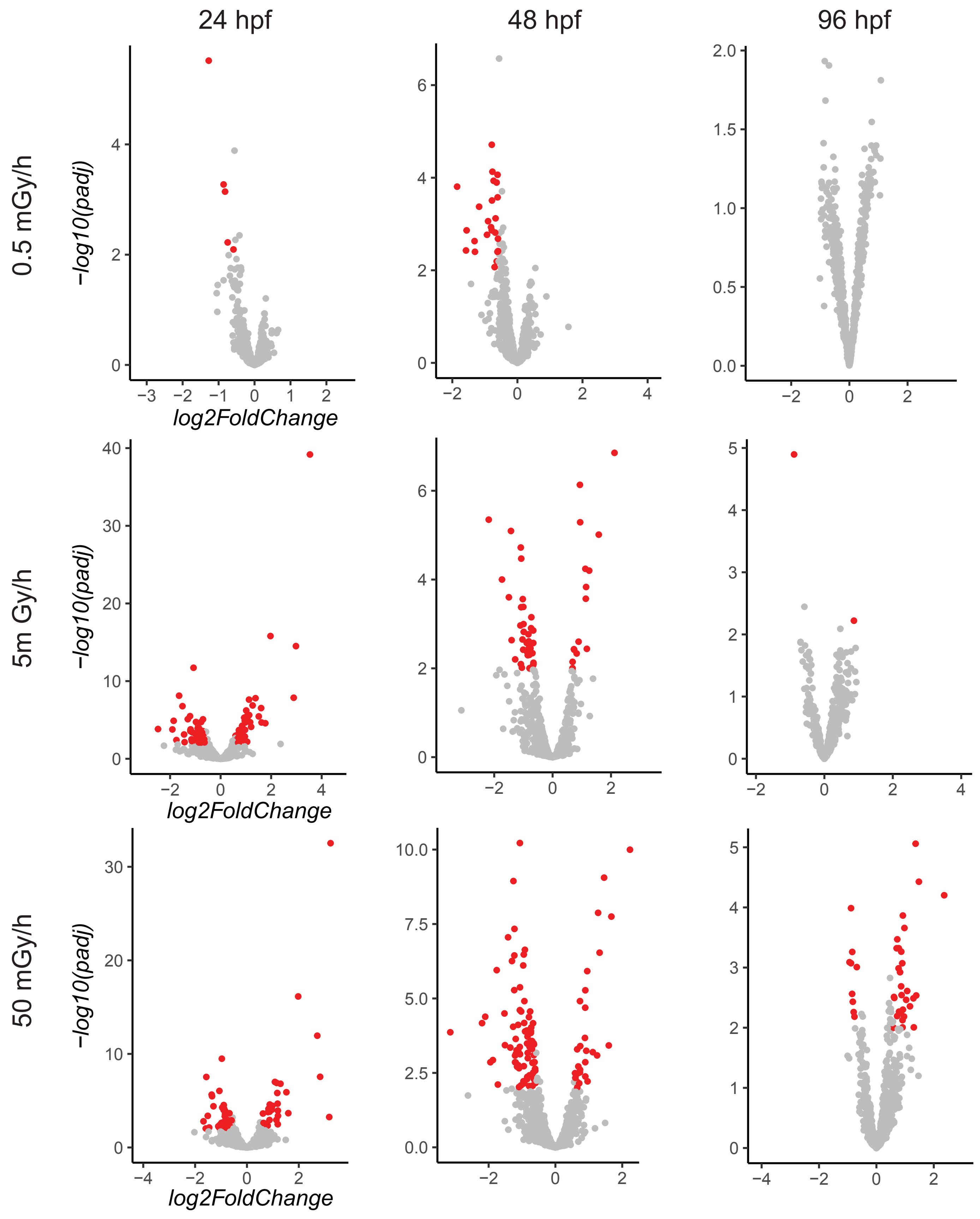

Supplementary Figure S7

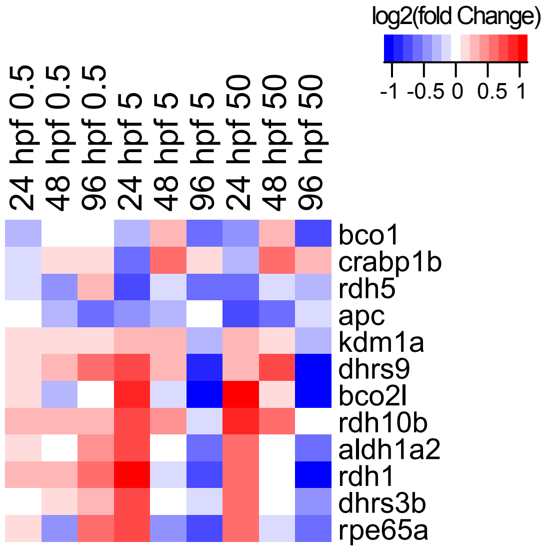

Supplementary Figure S8

Supplementary Table 1

Target= 50 mGy/h

Test	MCNP simulation 370 GBq (mGy/h)	MCNP sd	Corrected measure (mGy/h)	**Average dose rate (mGy/h)**	**sd**
1	63.1168	0.0035	48.22		
2	59.7533	0.0036	45.65		
3	60.6307	0.0036	46.32		
4	60.1735	0.0036	45.97		
5	61.884	0.0035	47.28		
6	61.9783	0.0035	47.35	**46.80**	**0.98**
7	61.3128	0.0037	46.84		
8	58.0429	0.0038	44.35		
9	58.8079	0.0038	44.93		
10	58.2608	0.0038	44.51		
11	60.1519	0.0038	45.96		
12	60.1714	0.0037	45.97	**45.43**	**0.98**

Target= 5 mGy/h

Test	MCNP simulation 370 GBq (mGy/h)	MCNP sd	Corrected measure (mGy/h)	**Average dose rate (mGy/h)**	**sd**
1	5.98406	0.0024	5.13		
2	5.98896	0.002	5.13		
3	6.02901	0.0029	5.16		
4	5.97657	0.0025	5.12		
5	5.98929	0.0025	5.13		
6	5.99333	0.0023	5.13	**5.13**	**0.02**
7	5.63259	0.0025	4.82		
8	5.6685	0.0029	4.86		
9	5.57349	0.0033	4.77		
10	5.66473	0.0036	4.85		
11	5.69105	0.0059	4.87		
12	5.56204	0.0024	4.76	**4.82**	**0.05**

Target= 0.5 mGy/h

Test	MCNP simulation 370 GBq (mGy/h)	MCNP sd	Corrected measure (mGy/h)	**Average dose rate (mGy/h)**	**sd**
1	0.519629	0.0144	0.49		
2	0.505474	0.004	0.48		
3	0.507387	0.0114	0.48		
4	0.512226	0.0099	0.48		
5	0.513982	0.0093	0.48		
6	0.513566	0.0119	0.48	**0.48**	**0.00**
7	0.47859	0.0121	0.45		
8	0.473933	0.0075	0.45		
9	0.463613	0.0051	0.44		
10	0.474284	0.0108	0.45		
11	0.480029	0.0102	0.45		
12	0.478105	0.013	0.45	**0.45**	**0.01**

Supplementary Table 2

Sample	Replicate	Dose rate mGy/h	Stage	Irradiation batch	Total R1	% > Q30 R1	Total R2	% > Q30 R2	Total
05mG_24h	Sample_I10_24h_05mG_E1	05mG	24h	I10	41913039	99.99	41913039	99.99	83826078
05mG_24h	Sample_I10_24h_05mG_E4	05mG	24h	I10	44401576	99.99	44401576	99.99	88803152
05mG_24h	Sample_I10_24h_05mG_E2	05mG	24h	I10	47242168	99.99	47242168	99.99	94484336
05mG_48h	Sample_I10_48h_05mG_E5	05mG	48h	I10	38798546	99.97	38798546	99.97	77597092
05mG_48h	Sample_I10_48h_05mG_E7	05mG	48h	I10	45576387	99.99	45576387	99.99	91152774
05mG_48h	Sample_I10_48h_05mG_E6	05mG	48h	I10	50573389	99.98	50573389	99.98	101146778
05mG_96h	Sample_I10_96h_05mG_E11	05mG	96h	I10	44243961	99.99	44243961	99.99	88487922
05mG_96h	Sample_I10_96h_05mG_E10	05mG	96h	I10	48320637	99.99	48320637	99.99	96641274
05mG_96h	Sample_I10_96h_05mG_E9	05mG	96h	I10	49384249	99.97	49384249	99.97	98768498
C_24h	Sample_I10_24h_C2	C	24h	I10	44723307	100	44723307	100	89446614
C_24h	Sample_I10_24h_C3	C	24h	I10	48322990	99.99	48322990	99.99	96645980
C_48h	Sample_I10_48h_C5	C	48h	I10	46734618	99.97	46734618	99.97	93469236
C_48h	Sample_I10_48h_C7	C	48h	I10	50826601	99.99	50826601	99.99	101653202
C_48h	Sample_I10_48h_C6	C	48h	I10	53318199	99.99	53318199	99.99	106636398
C_48h	Sample_I10_48h_C8	C	48h	I10	57294177	100	57294177	100	114588354
C_96h	Sample_I10_96h_C10	C	96h	I10	44078866	99.99	44078866	99.99	88157732
C_96h	Sample_I10_96h_C11	C	96h	I10	49202976	100	49202976	100	98405952
C_96h	Sample_I10_96h_C9	C	96h	I10	53973047	100	53973047	100	107946094
C_96h	Sample_I10_96h_C12	C	96h	I10	54142072	100	54142072	100	108284144
50mG_24h	Sample_I17_24h_50mG_E1	50mG	24h	I17	43129440	99.99	43129440	99.99	86258880
50mG_24h	Sample_I17_24h_50mG_E3	50mG	24h	I17	51508225	99.99	51508225	99.99	103016450
50mG_24h	Sample_I17_24h_50mG_E4	50mG	24h	I17	55599620	99.99	55599620	99.99	111199240
50mG_48h	Sample_I17_48h_50mG_E6	50mG	48h	I17	46960050	99.99	46960050	99.99	93920100
50mG_48h	Sample_I17_48h_50mG_E5	50mG	48h	I17	57311143	99.99	57311143	99.99	114622286
50mG_48h	Sample_I17_48h_50mG_E8	50mG	48h	I17	63127230	100	63127230	100	126254460
50mG_96h	Sample_I17_96h_50mG_E11	50mG	96h	I17	43547091	100	43547091	100	87094182
50mG_96h	Sample_I17_96h_50mG_E9	50mG	96h	I17	54400774	100	54400774	100	108801548
50mG_96h	Sample_I17_96h_50mG_E10	50mG	96h	I17	65403006	99.98	65403006	99.98	130806012
5mG_24h	Sample_I17_24h_5mG_E2	5mG	24h	I17	39467651	99.99	39467651	99.99	78935302
5mG_24h	Sample_I17_24h_5mG_E4	5mG	24h	I17	42248035	99.98	42248035	99.98	84496070
5mG_24h	Sample_I17_24h_5mG_E1	5mG	24h	I17	52362894	99.99	52362894	99.99	104725788
5mG_48h	Sample_I17_48h_5mG_E8	5mG	48h	I17	42512726	99.98	42512726	99.98	85025452
5mG_48h	Sample_I17_48h_5mG_E5	5mG	48h	I17	50556627	99.97	50556627	99.97	101113254
5mG_48h	Sample_I17_48h_5mG_E7	5mG	48h	I17	66495789	99.99	66495789	99.99	132991578
5mG_96h	Sample_I17_96h_5mG_E11	5mG	96h	I17	43832168	100	43832168	100	87664336
5mG_96h	Sample_I17_96h_5mG_E10	5mG	96h	I17	45062602	99.99	45062602	99.99	90125204
5mG_96h	Sample_I17_96h_5mG_E9	5mG	96h	I17	45474269	100	45474269	100	90948538
C_24h	Sample_I17_24h_C3	C	24h	I17	40480792	99.96	40480792	99.96	80961584
C_24h	Sample_I17_24h_C1	C	24h	I17	46119497	100	46119497	100	92238994
C_24h	Sample_I17_24h_C4	C	24h	I17	51310846	100	51310846	100	102621692
C_48h	Sample_I17_48h_C5	C	48h	I17	45260166	99.99	45260166	99.99	90520332
C_48h	Sample_I17_48h_C8	C	48h	I17	46988056	99.99	46988056	99.99	93976112
C_48h	Sample_I17_48h_C7	C	48h	I17	49184219	99.99	49184219	99.99	98368438
C_96h	Sample_I17_96h_C9	C	96h	I17	45107672	100	45107672	100	90215344
C_96h	Sample_I17_96h_C10	C	96h	I17	49587991	99.97	49587991	99.97	99175982
C_96h	Sample_I17_96h_C11	C	96h	I17	54422077	99.97	54422077	99.97	108844154
05mG_24h	Sample_I32_24h_0.5mG_E2	05mG	24h	I32	46924250	100	46924250	100	93848500
05mG_24h	Sample_I32_24h_0.5mG_E5	05mG	24h	I32	49716321	99.99	49716321	99.99	99432642
05mG_24h	Sample_I32_24h_0.5mG_E3	05mG	24h	I32	52121243	100	52121243	100	104242486
05mG_24h	Sample_I32_24h_0.5mG_E1	05mG	24h	I32	52826874	100	52826874	100	105653748
05mG_24h	Sample_I32_24h_0.5mG_E6	05mG	24h	I32	55481337	99.99	55481337	99.99	110962674
05mG_24h	Sample_I32_24h_0.5mG_E4	05mG	24h	I32	62317456	99.99	62317456	99.99	124634912
C_24h	Sample_I32_24h_C6	C	24h	I32	41854845	99.99	41854845	99.99	83709690
C_24h	Sample_I32_24h_C3	C	24h	I32	44201895	99.98	44201895	99.98	88403790
C_24h	Sample_I32_24h_C4	C	24h	I32	48031701	99.99	48031701	99.99	96063402
C_24h	Sample_I32_24h_C5	C	24h	I32	51135001	100	51135001	100	102270002
C_24h	Sample_I32_24h_C1	C	24h	I32	53116912	99.99	53116912	99.99	106233824
C_24h	Sample_I32_24h_C2	C	24h	I32	53225554	99.99	53225554	99.99	106451108

Supplementary Table 6

GO ID	GO Description	p-value	Ensembl geneID
GO:0042462	eye photoreceptor cell development	0.00043916	ENSDARG00000070107/ENSDARG00000011989/ENSDARG00000019566
GO:0001754	eye photoreceptor cell differentiation	0.00123495	ENSDARG00000070107/ENSDARG00000011989/ENSDARG00000019566
GO:0042461	photoreceptor cell development	0.00151927	ENSDARG00000070107/ENSDARG00000011989/ENSDARG00000019566
GO:0048666	neuron development	0.00183896	ENSDARG00000070107/ENSDARG00000011989/ENSDARG00000093357/ENSDARG00000017007/ENSDARG00000019566/ENSDARG00000045639/ENSDARG00000103937
GO:0044262	cellular carbohydrate metabolic process	0.00184156	ENSDARG00000074684/ENSDARG00000105039/ENSDARG00000100352
GO:0005977	glycogen metabolic process	0.00274645	ENSDARG00000105039/ENSDARG00000100352
GO:0006073	cellular glucan metabolic process	0.00274645	ENSDARG00000105039/ENSDARG00000100352
GO:0044042	glucan metabolic process	0.00274645	ENSDARG00000105039/ENSDARG00000100352
GO:0044264	cellular polysaccharide metabolic process	0.00332231	ENSDARG00000105039/ENSDARG00000100352
GO:0043401	steroid hormone mediated signaling pathway	0.00354339	ENSDARG00000017007/ENSDARG00000004861/ENSDARG00000031768
GO:0046530	photoreceptor cell differentiation	0.00354339	ENSDARG00000070107/ENSDARG00000011989/ENSDARG00000019566
GO:0005976	polysaccharide metabolic process	0.00362957	ENSDARG00000105039/ENSDARG00000100352
GO:0006112	energy reserve metabolic process	0.00362957	ENSDARG00000105039/ENSDARG00000100352
GO:0071383	cellular response to steroid hormone stimulus	0.00367306	ENSDARG00000017007/ENSDARG00000004861/ENSDARG00000031768
GO:0005975	carbohydrate metabolic process	0.00372343	ENSDARG00000074684/ENSDARG00000002197/ENSDARG00000013721/ENSDARG00000105039/ENSDARG00000100352
GO:0006097	glyoxylate cycle	0.00392107	ENSDARG00000074684
GO:0009755	hormone-mediated signaling pathway	0.00579812	ENSDARG00000017007/ENSDARG00000004861/ENSDARG00000031768
GO:0048545	response to steroid hormone	0.00579812	ENSDARG00000017007/ENSDARG00000004861/ENSDARG00000031768
GO:0071407	cellular response to organic cyclic compound	0.00597272	ENSDARG00000017007/ENSDARG00000004861/ENSDARG00000031768
GO:0010623	programmed cell death involved in cell development	0.00782702	ENSDARG00000011989
GO:0019918	peptidyl-arginine methylation, to symmetrical-dimethyl arginine	0.00782702	ENSDARG00000033134
GO:0043576	regulation of respiratory gaseous exchange	0.00782702	ENSDARG00000027529
GO:0045576	mast cell activation	0.00782702	ENSDARG00000010420
GO:0046487	glyoxylate metabolic process	0.00782702	ENSDARG00000074684
GO:0046666	retinal cell programmed cell death	0.00782702	ENSDARG00000011989
GO:0046668	regulation of retinal cell programmed cell death	0.00782702	ENSDARG00000011989
GO:0046671	negative regulation of retinal cell programmed cell death	0.00782702	ENSDARG00000011989
GO:0110111	negative regulation of animal organ morphogenesis	0.00782702	ENSDARG00000011989
GO:0071396	cellular response to lipid	0.00831568	ENSDARG00000017007/ENSDARG00000004861/ENSDARG00000031768
GO:0048562	embryonic organ morphogenesis	0.00952709	ENSDARG00000070107/ENSDARG00000040606/ENSDARG00000078052/ENSDARG00000062262/ENSDARG00000103937

Supplementary Table 7

UniProtKB/ TrEMBL ID	*UniProtKB gene name*	*log2(fold change)*	*-log10(pval)*	*Zfin id*
A0A0N9P0E6	opn1lw2	-0.93	3.14	opn1lw2
A0A0R4I9C3	rbm42	1.17	2.26	rbm42
A0A0R4IS09	col14a1b	-1.95	2.36	col14a1b
A0A2R8PVL6	timm44	1.41	3.50	timm44
A0A2R8Q8V9	eml4	-0.64	2.65	NA
A0A2R8QTL0	mylpfa	-0.79	3.48	mylpfa
A0A2R8QTM1	cand2	-1.88	2.33	cand2
A0A2R8QV67	vtg4	0.67	4.21	vtg4
A0A2R8RI79	NA	1.33	2.54	si:zfos-1505d6.3
A0A2R8RQJ2	NA	1.90	2.48	NA
A0A2R8RX88	fus	0.92	2.97	fus
A2BFS9	tcnbb	0.98	2.27	tcnbb
A5WWB7	mybpc1	-1.22	2.59	mybpc1
A7E2K5	crybb1l2	-0.65	4.47	crybb1l2
A7MCR8	NA	1.80	2.86	si:dkey-26g8.5
A8KBU7	mybphb	-0.72	2.04	mybphb
A8WG31	NA	1.69	2.89	NA
B0V0U4	galnt2	-1.44	2.60	galnt2
B8A518	tuba1c	-0.76	2.74	tuba1c
E7F351	acsl2	-0.76	2.51	acsl2
E7F8M1	crybb1l1	-0.59	4.37	crybb1l1
E7FFZ3	NA	0.95	2.26	si:dkey-46g23.2
E9QDR6	NA	-0.62	2.22	si:ch211-183d21.1
E9QF07	myh7bb	-1.04	2.33	myh7bb
F1Q699	mybpc2b	-0.90	2.66	mybpc2b
F1QCK4	ryr1a	-1.18	2.63	ryr1a
F1QF69	soul5	0.69	2.49	soul5
F1QIP9	NA	1.18	3.26	zgc:165518
F1QVM8	cep350	-1.68	2.71	cep350
F1QW04	tcirg1b	-0.98	2.51	tcirg1b
F8W5L3	oat	-1.44	3.08	oat
M1EVR1	SLC12A5	-1.34	2.69	NA
Q08C39	mrps5	-1.24	2.02	NA
Q08CP3	neo1a	-1.45	2.77	NA
Q32Q50	map2k2a	-1.48	2.00	NA
Q4V9E0	fmoda	-1.94	2.68	NA
Q508P8	smyhc1	-0.96	3.21	NA
Q52JI7	cryba4	-0.63	4.71	cryba4
Q5PR28	bfsp2	-1.38	2.13	NA
Q5RIL8	hsd3b2	-2.85	2.83	hsd3b2
Q5U373	cab39l	0.71	2.69	cab39l
Q6DGY4	cryba1b	-0.64	3.68	cryba1b
Q6NY70	epcam	0.62	2.85	NA
Q6PBN3	prph2b	-0.70	3.28	prph2b
Q6XNL8	acta1b	-0.59	2.06	acta1b
Q7SZM5	mmp2	-0.97	2.33	NA
Q7ZTV9	alpl	-0.91	4.65	NA
Q7ZTX2	wdr5	-1.18	2.21	wdr5
Q7ZTZ7	bckdk	1.04	2.04	NA
Q7ZUB0	myl13	-0.65	2.03	myl13
Q8UUZ6	cryaa	-1.28	2.19	cryaa
Q90WT1	crybb1	-0.59	3.41	crybb1
Q9PU59	nipsnap1	-2.01	2.45	NA
R4GE02	NA	0.60	3.91	si:ch211-113a14.11
X1WC82	rps28	-0.62	2.12	rps28
X1WEZ4	col6a4a	-0.85	2.12	col6a4a

Supplementary Table 8

UniProtKB/	*UniProtKB*	*log2(fold change)*	*-log10(pval)*	*Zfin id*
A0A0A0VG08	mt-co2	-0.75	3.19	mt-co2
A0A0N9P0E6	opn1lw2	-0.97	3.73	opn1lw2
A0A0R4IA33	nots	-0.63	2.05	nots
A0A0R4IRS1	ap1m1	-1.03	2.04	ap1m1
A0A0R4ISJ3	xrn2	-0.87	3.16	xrn2
A0A0R4IWP3	psmb2	-2.35	3.06	psmb2
A0A140LGG1	plxna1a	-1.16	2.40	plxna1a
A0A140LGT2	cpeb4a	-0.81	2.13	cpeb4a
A0A140LH63	si	0.73	3.98	i:ch73-368j24.12
A0A2R8Q5Z6	actn3a	0.65	4.77	actn3a
A0A2R8Q7K6	vil1	-0.71	4.17	vil1
A0A2R8QAE1	LOC100334800	-0.98	2.14	NA
A0A2R8QBD4	ythdf2	-1.17	2.80	ythdf2
A0A2R8QFA6	timm8a	-0.77	2.15	timm8a
A0A2R8QKL9	tgm1l1	0.69	3.77	tgm1l1
A0A2R8QMT2	wdr7	-1.17	2.23	NA
A0A2R8QP59	stxbp1b	-0.62	2.00	stxbp1b
A0A2R8QPB1	gad1b	-1.16	2.62	gad1b
A3KNH4	plod3	0.79	2.15	plod3
A3KQL1	necab2	-0.64	2.05	necab2
A4JYG9	ncam2	-5.25	2.74	NA
A5PF53	arhgap35b	-0.90	2.09	arhgap35b
A5PN62	ddah2	-1.42	2.09	ddah2
A5X6X5	ttn.2	0.88	7.95	NA
A6H8Q2	crtac1a	1.18	2.46	NA
A7E2J3	NA	-1.11	2.50	NA
A8E560	atp6v0d1	-0.88	2.64	NA
A8E7L6	nudcd1	-1.30	3.56	nudcd1
A8KBU7	mybphb	0.85	5.24	mybphb
B0UXI4	syn1	-0.62	2.22	syn1
B2GS08	actb1	-2.36	2.75	NA
B3DIE8	tpp1	-0.65	4.02	NA
B8A4G3	ppm1aa	-0.94	2.51	ppm1aa
B8A569	myhz1.3	1.87	4.81	myhz1.3
B8A611	engase	-1.17	2.32	engase
E7EZ79	mybpc3	0.77	2.50	mybpc3
E7F0E2	gnpda2	-2.43	2.05	gnpda2
E7F2X5	itga1	0.80	2.73	itga1
E7F3A6	tcerg1b	-1.58	2.76	NA
E9QDR6	NA	-1.10	2.47	i:ch211-183d21.1
F1Q699	mybpc2b	0.90	5.63	mybpc2b
F1Q7I4	b3glcta	0.60	4.97	b3glcta
F1QF69	soul5	0.88	3.52	soul5
F1QYS7	acss1	-1.23	2.99	acss1
F1R4W2	lgals3a	-1.61	2.80	lgals3a
F1R7N8	ttn.2	1.05	3.21	ttn.2
F1R8W4	desma	0.60	6.11	desma
F1RAM4	NA	-0.68	2.81	zgc:110339
G1K2X0	ttn.1	0.83	6.94	ttn.1
G8IHU0	lama2	0.60	3.93	NA
Q07DR3	dctn1a	-1.72	2.14	NA
Q0P414	NA	2.50	5.51	NA
Q1LVH7	celf5a	-0.84	2.28	celf5a
Q568F8	ssbp1	-2.17	4.79	NA
Q588C3	dnmt3ba	0.59	4.30	NA
Q5NJL3	matn3a	0.96	5.68	NA
Q5RHK5	mtmr9	-1.99	3.39	mtmr9
Q5RIG8	srgap1b	1.83	2.24	srgap1b
Q5TYT1	nrcama	-1.30	2.06	nrcama
Q5U3E5	slc6a1b	-1.50	2.73	slc6a1b
Q5XJM8	wdfy1	0.67	2.56	NA
Q68EI7	fuca2	1.80	2.14	NA
Q6DRM4	parvaa	-1.26	3.90	parvaa

Q6IQE4	tnni2a.4	0.99	4.48	NA
Q6NZW3	cfl2	-1.01	2.37	cfl2
Q6P1J5	atp5f1d	-0.67	3.38	NA
Q6PI45	igbp1	-0.92	2.73	NA
Q6ZM52	sart3	-1.00	2.13	sart3
Q7T1B1	hbbe3	-0.61	4.01	NA
Q7T1R2	tgfbi	1.66	2.04	NA
Q7T2E6	xab2	-1.76	2.83	NA
Q7ZT36	pvalb3	-1.24	2.66	pvalb3
Q7ZTZ7	bckdk	1.21	2.48	NA
Q7ZUB0	myl13	0.69	3.24	myl13
Q7ZV01	NA	-1.76	2.10	NA
Q7ZV33	tm9sf3	1.27	2.06	NA
Q7ZVJ0	pfn2l	-0.83	3.23	pfn2l
Q7ZVP3	matn1	2.34	3.44	NA
Q7ZVX4	rab14	-0.59	3.52	rab14
Q7ZVX8	atp6v1d	-1.59	3.29	atp6v1d
Q804W0	pvalb1	-0.61	2.04	pvalb1
Q90YJ0	col1a2	0.66	5.09	NA
Q9DDS7	cd81a	-0.73	2.45	NA
Q9I8U9	tnnt3a	0.83	3.72	tnnt3a
X1WDH8	NA	0.70	2.33	i:dkey-261m9.19
X1WGK9	NA	0.68	6.83	zgc:153405
X4YKC7	CRP	-1.64	4.63	NA
Z4YJ68	cisd2	-1.03	3.57	cisd2
